# The impact of community-delivered models of malaria control and elimination: a systematic review

**DOI:** 10.1186/s12936-019-2900-1

**Published:** 2019-08-06

**Authors:** Win Han Oo, Lisa Gold, Kerryn Moore, Paul A. Agius, Freya J. I. Fowkes

**Affiliations:** 1 School of Health and Social Development, Deakin University, 221 Burwood Hwy, Burwood, VIC 3125 Australia; 2 Burnet Institute, 85 Commercial Rd, Melbourne, VIC 3004 Australia; 3 Melbourne School of Population and Global Health, The University of Melbourne, 235 Bouverie St, Carlton, Melbourne, VIC 3053 Australia; 4 Department of Epidemiology and Preventive Medicine, Monash University, Melbourne, VIC Australia; 5 Judith Lumley Centre, La Trobe University, Level 3, George Singer Building Bundoora, Melbourne, VIC 3086 Australia

**Keywords:** Malaria, Community health worker, Community-delivered model, Coverage, Malaria-metric outcomes

## Abstract

**Background:**

Community-delivered models have been widely used to reduce the burden of malaria. This review aimed to explore different community-delivered models and their relative effectiveness in terms of coverage and malaria-metric outcomes in order to inform the design and implementation of Community Health Worker (CHW) programmes for malaria control and elimination.

**Methods:**

A systematic review of studies investigating the impact of community-delivered models on coverage and malaria-metric (parasitaemia and hyperparasitaemia, malaria case and mortality, anaemia, and fever) outcomes compared to non- community-delivered models was undertaken by searching in five databases of published papers and grey literature databases. Data were extracted from studies meeting inclusion and quality criteria (assessed using relevant tools for the study design) by two independent authors. Meta-analyses were performed where there was sufficient homogeneity in effect and stratified by community-delivered models to assess the impact of each model on coverage and malaria-metric outcomes.

**Results:**

28 studies were included from 7042 records identified. The majority of studies (25/28) were performed in high transmission settings in Africa and there was heterogeneity in the type of, and interventions delivered as part of the community-delivered models. Compared to non- community-delivered models, community-delivered models increased coverage of actual bed net usage (Relative Risk (RR) = 1.64 95% CI 1.39, 1.95), intermittent preventive treatment in pregnancy (RR = 1.36 95% CI 1.29, 1.44) and appropriate and timely treatment of febrile children, and improved malaria-metric outcomes such as malaria mortality (RR = 0.58 95% CI 0.52, 0.65). However, the considerable heterogeneity was found in the impact of community-delivered models in reducing, parasitaemia and hyperparasitaemia prevalence, anaemia incidence, fever prevalence and malaria caseload. Statistical comparisons of different community-delivered models were not undertaken due to the heterogeneity of the included studies in terms of method and interventions provided.

**Conclusion:**

Overall, the community-delivered model is effective in improving the coverage of malaria interventions and reducing malaria-associated mortality. The heterogeneity of the community-delivered models and their impact on malaria-metric indices suggests that evidence for context-specific solutions is required. In particular, community-delivered models for malaria elimination, integrated with services for other common primary health problems, are yet to be evaluated.

**Electronic supplementary material:**

The online version of this article (10.1186/s12936-019-2900-1) contains supplementary material, which is available to authorized users.

## Background

Malaria caused by *Plasmodium* spp. is an infectious disease transmitted by Anopheles mosquitoes. Despite a 18% decrease in malaria incidence rate since 2010 [1] and reinvigorated goals of malaria elimination, in the past few years the decline in the malaria burden has stalled and around half the global population is still at risk of malaria with around 219 million cases and 435,000 deaths in 2017 [1]. Recent reductions in malaria incidence has largely been attributed to the introduction of highly efficacious artemisinin-based combination therapy and high universal coverage of long-lasting insecticidal nets (LLIN), as well as testing (through increased availability and accessibility of rapid diagnostic tests) and targeted treatment of at-risk populations [2, 3].

A key challenge in malaria control is delivery of malaria interventions to the communities that need them most. One widely used model is a community-delivered model in which malaria interventions are delivered by community health workers (CHW). The World Health Organization defines that CHW should be members of the communities where they work, selected by, and answerable to, the communities, supported by the health system, but not necessarily a part of its organization, and should have shorter training than professional workers [4]. The use of CHW is attractive as they can be implemented with minimal training [5], they are readily available and are effective and cost-effective in resource-limited countries and several health areas [6–8].

Different community-delivered models for malaria control and elimination have been developed and implemented, namely the traditional CHW (tCHW) model, Integrated Community Case Management (iCCM), and Home Management of Malaria (HMM) according to the services provided and level of delegation of the interventions (Table 1). The tCHW model has been widely used since the 1980s across Africa and Asia to deliver malaria services. In the tCHW model, health authorities train the community-selected volunteers and equip them with tools such as malaria diagnostic tests, anti-malarial medicines, and communication tools for behaviour change communication for malaria prevention and control. The volunteers then provide malaria services for their community with the supervision and support of health staff. iCCM is a more recent strategy adopted in 2010 and has been used in some African countries, such as Nigeria and Uganda. The iCCM is an integrated model, intended for children under 5 years old, whereby malaria interventions are delivered along with the interventions for common life-threatening non-malaria diseases, typically pneumonia and diarrhoea. HMM has been implemented in many African countries including Uganda, Senegal, Nigeria, Ghana, Guinea, Ethiopia, Burkina Faso and Cameroon, and is characterized as deployment of malaria diagnosis, treatment and referral services up to the household level and in this model, the household member (usually the mothers or caregivers of under-5 children) prescribes anti-malarial medicine to other household members or neighbours (usually the under-5 children of own household or neighbours’ households).

**Table 1:** Characteristics of the 28 included papers

Author	Country	Malaria transmission	Design	Model	Malaria (M) or malaria plus (M+)	Malaria intervention delivered
Linn et al. [36]	Myanmar	Endemic^a^	Retrospective cohort	tCHW	M	Parasitological dx + Tx + referral + BCC
Alonso et al. [57]	Gambia	Holo-endemic^a^	Quasi-experiment with control	tCHW	M	ITN + parasitological dx + Tx + referral + BCC
Delacollette et al. [40]	Zaire (Congo)	Meso-endemic^a^	Quasi-experiment with control	tCHW	M	ITN + chemoprohylaxis
Greenwood et al. [41]	Gambia	Seasonal^a^	Quasi-experiment with control	tCHW	M	IPTc
Hamainza et al. [46]	Zambia	Perennial^a^	Quasi-experiment with control	tCHW	M	ITN + parasitological dx + Tx + referral
Linn et al. [44]	Senegal	Pf API ≥ 0.1/1000 pa^b^	Quasi-experiment with control	tCHW	M	Parasitological dx + Tx + referral + BCC
Littrell et al. [35]	Cameroon	Endemic and perennial^a^	Quasi-experiment with control	tCHW	M	Parasitological dx + Tx + referral
Mbonye et al. [56]	Uganda	Hyper-endemic^a^	Quasi-experiment with control	tCHW	M	Presumptive dx + Tx + referral
Mbonye et al. [32]	Uganda	Hyper-endemic^a^	Quasi-experiment with control	tCHW	M	Presumptive dx + Tx + referral
Msyamboza [47]	Malawi	Pf API ≥ 0.1/1000 pa^b^	Quasi-experiment with control	tCHW	M	ITN + IRS + parasitological dx + Tx + referral
Ndyomugyenyi et al. [58]	Uganda	Pf API ≥ 0.1/1000 pa^b^	Quasi-experiment with control	tCHW	M	Presumptive dx + Tx + referral
Tiono et al. [45]	Burkina Faso	Seasonal^a^	Quasi-experiment with control	tCHW	M	Parasitological dx + Tx
Das et al. [26]	India	API > 5/1000 pa^a^	Cluster randomized trial	tCHW	M	Presumptive dx + Tx + chemoprophylaxis
Eriksen et al. [49]	Tanzania	Holo-endemic^a^	Cluster randomized trial	tCHW	M	IPTp + BCC
Kweku et al. [34]	Ghana	Pf API ≥ 0.1 per 1000 pa^b^	Cluster randomized trial	tCHW	M	Presumptive dx + Tx + referral + BCC
Lemma et al. [43]	Ethiopia	Hypo-endemic^a^	Cluster randomized trial	tCHW	M	ITN + BCC
Ohnmar et al. [30]	Myanmar	Endemic^a^	Cluster randomized trial	tCHW	M	Parasitological dx + Tx + referral
Patouillard et al. [33]	Ghana	Seasonal^a^	Cluster randomized trial	tCHW	M	ITN + presumptive dx + Tx + referral + BCC
Abegunde et al. [27]	Nigeria	High perennial^a^	Case–control study	iCCM	M+^c^	ITN + parasitological dx + Tx + referral + BCC + IPTp
Brenner et al. [28]	Uganda	Pf API ≥ 0.1 per 1000 pa^b^	Quasi-experiment with control	iCCM	M+^d^	Parasitological dx + Tx + referral + BCC
Mubiru et al. [38]	Uganda	Pf API ≥ 0.1 per 1000 pa^b^	Quasi-experiment with control	iCCM	M+^d^	Parasitological dx + Tx + referral + BCC
Nsungwa-Sabiiti et al. [37]	Uganda	Holo-endemic to hyper-endemic^a^	Quasi-experiment with control	HMM	M+^d^	Parasitological dx + Tx + referral + BCC
Thiam et al. [42]	Senegal	Pf API ≥ 0.1 per 1000 pa^b^	Quasi-experiment with control	HMM	M+^d^	Presumptive dx + Tx + referral + BCC
Tobin-West et al. [31]	Nigeria	Pf API ≥ 0.1 per 1000 pa^b^	Quasi-experiment with control	HMM	M+^e^	Parasitological dx + Tx + referral + BCC
Chinbuah et al. [48]	Ghana, Guinea	Perennial^a^	Cluster randomized trial	HMM	M+^f^	Presumptive dx + Tx + referral + BCC
Kidane and Morrow [59]	Ethiopia	Hyper-endemic^a^	Cluster randomized trial	HMM	M	Presumptive dx + Tx + referral + BCC
Kouyaté et al. [39]	Burkina Faso	Holo-endemic^a^	Cluster randomized trial	HMM	M	Parasitological dx + Tx + referral + BCC
The CDI Study Group [29]	Cameroon, Nigeria and Uganda	Pf API ≥ 0.1 per 1000 pa^b^	Cluster randomized trial	HMM	M+^g^	ITN, presumptive dx + Tx

pa, per annum; BCC, behavioural change communication/health education activities; Chemoprophylaxis, provision of malaria chemoprophylaxis; IPTc, intermittent preventive treatment (children); IPTp, intermittent preventive treatment (pregnant mothers); IRS, indoor residual spraying; ITN, long-lasting insecticidal nets or insecticide treated net distribution and/or organization for distribution by CHW and/or net dipping; Presumptive dx, malaria diagnosis by clinical signs and symptoms by CHWs; Parasitological dx, malaria diagnosis by a parasitological method either rapid diagnostic test, microscopy or polymerase chain reaction or others by CHWs; Referral, referral of malaria cases as per the guidelines; Tx, malaria treatment by CHWs

^a^Self-reported in the paper

^b^Derived from Malaria Atlas Project [21] Map

^c^Primary health care

^d^Pneumonia, diarrhoea

^e^Pneumonia, diarrhoea, malnutrition

^f^Diarrhoea

^g^Vitamin A supplementation, Short-course, directly-observed treatment for Tuberculosis, Ivermectin distribution

CHW will be essential in order to accelerate progress in reducing the burden of malaria and to achieve malaria elimination targets. As such, it is important to understand the utility and effectiveness of community-delivered models, which have varied across settings. Previous systematic reviews on community-delivered models, published between 2007 and 2014 [9–18], have been restricted to specific targeted population such as under-5 children [9, 10] or pregnant women [11, 18], interventions for malaria alone [12–14, 16, 17] or geographical regions such as sub-Saharan Africa [9, 10, 12, 15, 16] or low and middle income countries [11, 13]. The aim of this systematic review was to provide holistic evidence for the different types of community-delivered models and their relative effectiveness in changing coverage (community utilization of quality malaria services) and malaria-metric indices (malaria disease burden and its consequences), compared to non- community-delivered models, in order to inform the design and implementation of CHW for malaria control and elimination.

## Methods

A systematic review of published and grey literature of quantitative studies investigating community-delivered models for malaria only or malaria plus other diseases interventions to control or eliminate malaria was conducted. The terminology for community-delivered health care models varied across studies (Additional file 1) and is hereafter referred to as CHW. The protocol for this review was prospectively registered with PROSPERO (protocol number CRD42016052929) and adheres to the PRISMA [19] and MOOSE [20] guidelines for systematic reviews. The full protocol and data extraction tools can be found in Additional file 2 and is summarized below.

### Search strategy and selection criteria

**Fig. 1:**
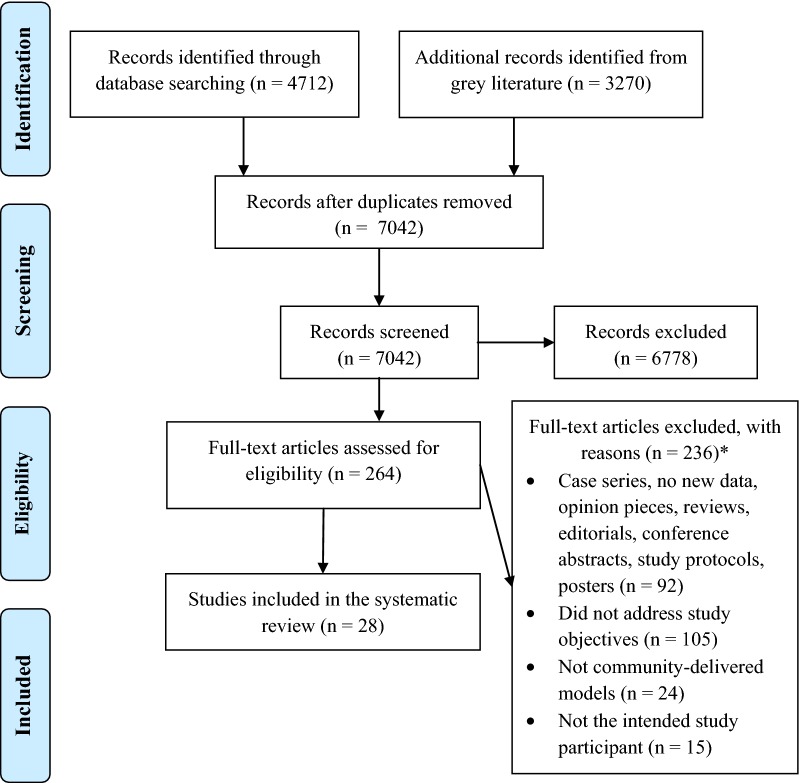
PRISMA 2009 flow diagram [55]. *Excluded papers with reasons are available in Additional file 4

All interventional and observational studies conducted on intended study participants in malaria-endemic areas (defined by the Malaria Atlas Project [21]) that contain data describing the outcomes or processes involved in community-based delivery of any malaria or malaria plus other diseases interventions were included (Fig. 1).

PubMed, Embase, Cochrane Central Register of Controlled Trials, LILACS and African Medicus Index were searched with no restrictions on language (Additional file 3) up to and including 6th September 2018. Searches included peer-reviewed publications and grey literature such as evaluation reports, policy guidelines and strategy documents (Additional file 3). The reference lists of the included papers were also reviewed. The first author (WHO) independently screened studies for inclusion (Fig. 1).

### Data extraction and analysis

Two independent authors (WHO and KM) extracted data using a proforma for quantitative synthesis (Additional file 2). Discrepancies were resolved through discussion with a third author (FJIF). If further information was required that was not featured in the literature, authors were contacted up to two times via email.

The outcomes of interest were malaria intervention coverage (number and/or percentage, or other measures of malaria service uptake such as bed net ownership (insecticide-treated net (ITN) ownership ≥ 1/household) and actual usage (ITN use (the previous night) by anyone in the household), intermittent preventive treatment in pregnancy (IPTp) coverage (pregnant women who completed two doses of sulfadoxine-pyrimethamine), appropriate and timely (treatment within 24 h after onset of fever) treatment for fever of under-5 children) and malaria metric indices (number and/or percentage of malaria infection (hyperparasitaemia defined as parasite density 7000/μl and parasitaemia as 5000/μl and over, or more than or equal to 2000 asexual forms of *Plasmodium falciparum* per mm^3^ in the blood detected by microscopy), cases and death, fever (body temperature more than 37.5 °C or reported fever cases) and anaemia (Hb < 8.0 g/dl or haematocrit ≤ 24%)).

Quality assessments were conducted by the first author (WHO). Risk of bias in non-randomized quantitative studies was assessed using The Risk Of Bias In Non-randomized Studies-of Interventions (ROBINS-I) assessment tool [22]. For randomized studies, The Cochrane Collaboration’s tool for assessing risk of bias [23] was used.

Meta-analyses were performed to obtain pooled estimates of each outcome overall and by community-delivered model strata (iCCM, HMM and tCHW). Between-study heterogeneity was assessed using the I-squared value [24]. Pooled estimates for each outcome were calculated using either a fixed-effects (where I-squared ≤ 30%) or random-effects model (I-squared = 31–75%). Results were not pooled if heterogeneity was high (I-squared > 75%). In the fixed-effects models, pooled effects estimates were weighted by the inverse of the individual study standard error. In random-effects models, a between-study variance component was incorporated into the study weights [25]. Meta-analyses calculated relative differences in risk (RR) based on the absolute numbers extracted from each study and using the formula (intervention numerator/intervention denominator)/(control numerator/control denominator) between intervention populations (CHW provided interventions) and comparator/control populations (other service provider provided interventions) in the follow up phases of experimental studies. Meta-analyses were stratified by community-delivered models and presented in forest plots for each unique coverage and impact indicator. The coverage and impact indicators were defined according to the defined and measured outcomes in the included studies. Due to the small number of papers included in the meta-analyses, sensitivity analyses were not done. All analyses were undertaken using the Stata version 13 statistical software package (V13; StataCorp, College Station, TX, USA).

## Results

In total, 7042 unique records were identified from five databases and grey literature searches. After screening of titles and abstracts, 264 full-text articles were assessed for eligibility, and of these 28 quantified the impact of community-delivered models on coverage and malaria-metric indices and were included in the quantitative data synthesis (Fig. 1).

Table 1 summarizes the 28 included studies (tCHW = 18, iCCM = 3 and HMM = 7) which investigated community-delivered malaria interventions. In most studies (n = 21), CHW provided malaria diagnosis, treatment and referral, whereas the remaining 7 studies provided IPT or bed net distributions by CHW. CHW only provided services for malaria in tCHW and HMM models; additional diagnosis, treatment and referral services for other diseases from CHW were available in the iCCM model. The majority of studies were conducted in Africa (n = 25), mostly in hyper/holoendemic settings, and 3 studies were conducted in Asia, which were typically performed in hypo/mesoendemic settings. Study designs were cluster-randomized trials (n = 10), quasi-experimental studies with control (n = 16), case control studies (n = 1) and retrospective cohort study (n = 1). Risk of bias was largely assessed as moderate for randomised studies (10/10) and moderate-to-serious for non-randomized studies (16/18) (Additional file 5). 15 studies reported coverage outcomes and 13 studies reported malaria-metric outcomes of CHW compared to non-community-delivered models.

### Impact of community-delivered models on coverage of malaria interventions

#### Bed net coverage

**Fig. 2:**
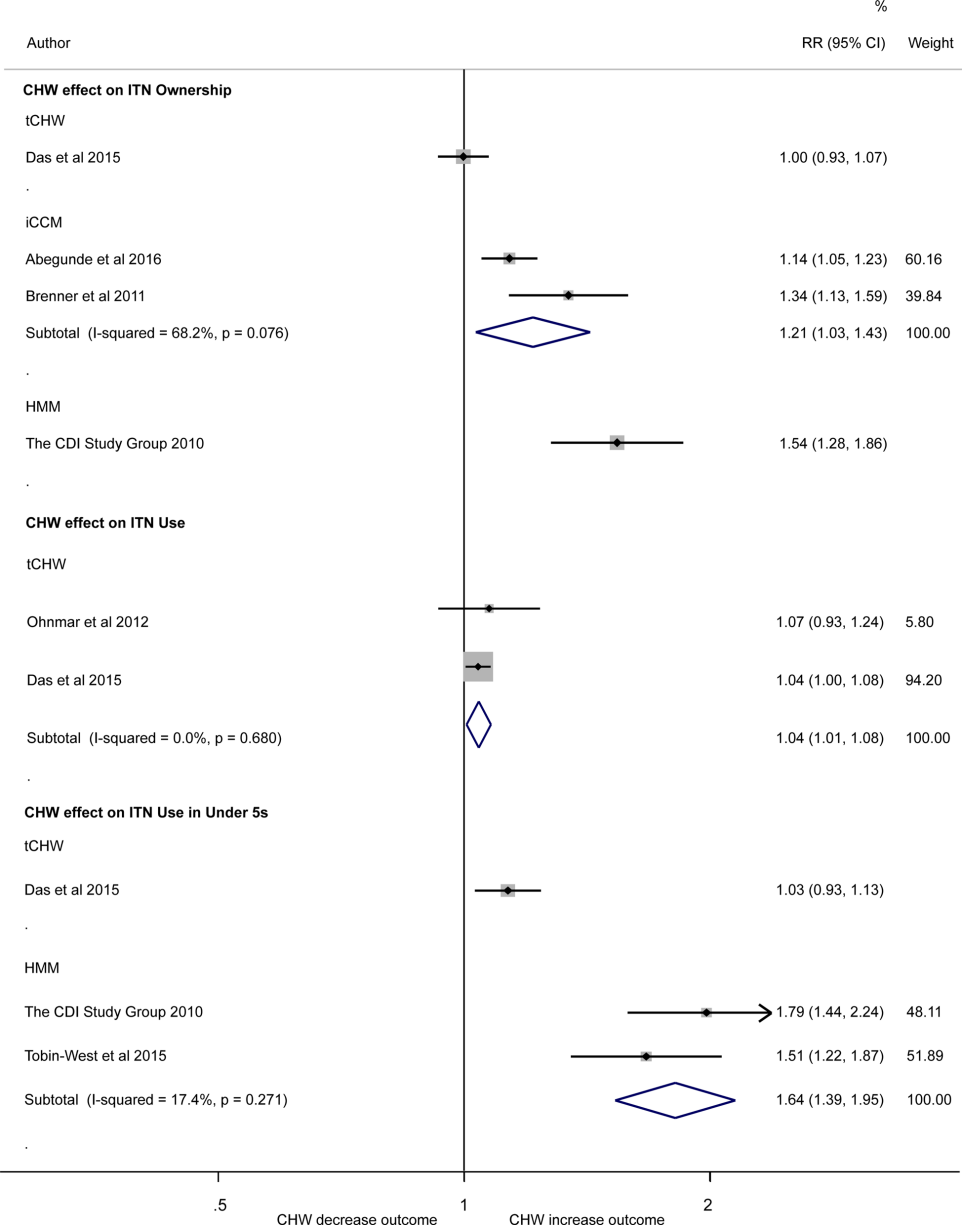
Forest plot showing the association between use of CHW and coverage of insecticide treated bed net ownership and use. Plot shows risk ratios (RR), 95% confidence intervals (95% CI) and inverse variance study weights (% weight). Pooled results were calculated by fixed-effects (I-squared ≤ 30%) or random-effects (I-squared = 31% to ≤ 75%). Estimates were calculated from data in the papers. ITN ownership is defined as a household with at least one ITN, ITN use as ITN use (the previous night) by anyone and ITN use (the previous night) by under-5 children. Please note: results were not pooled in meta-analysis across different community-delivered models quantifying the effect of community-delivered models on ITN ownership or ITN use (the previous night) by under-5 children due to the high degree of heterogeneity (I-squared = 88.6% and 93.2%, respectively)

There was considerable heterogeneity (I-squared = 88.6%) in 4 studies quantifying the effect of community-delivered models on household bed net ownership (ITN ownership ≥ 1/household) compared to non community-delivered models (interventions provided by non-CHW service providers) so results were not pooled. Estimates ranged from no association in one study investigating tCHW (RR = 1.00; 95% CI 0.93, 1.07) [26], to 1.14 times higher (95% CI 1.05, 1.23) [27] and 1.34 times higher (95% CI 1.13, 1.59) [28] risk of bed net ownership in two studies investigating iCCM, to a 1.54 (95% CI 1.28, 1.86) times higher risk in one study [29] investigating HMM compared to the non-CHW arms (the control) (Fig. 2).

Meta-analysis of two studies [26, 30] showed positive association between tCHW models of ITN distribution on ITN use (the previous night) by anyone in the household (pooled RR = 1.04 95% CI 1.01, 1.08; I-squared = 0%) compared to the non-CHW arm (Fig. 2). There was considerable heterogeneity in three studies quantifying the effect of community-delivered models on ITN use (the previous night) by under-5 children compared to non- community-delivered model (I-squared = 93.2%). Estimates ranged from no association in one study investigating tCHW (RR = 1.03; 95% CI 0.93, 1.13) [26], 1.51 times (95% CI 1.22, 1.87) [31] and 1.79 times (95% CI 1.44, 2.24) [29] higher risk of under-5 ITN use in HMM arm compared to the non-CHW arm (Fig. 2).

#### IPT coverage in pregnant women and children

**Fig. 3:**
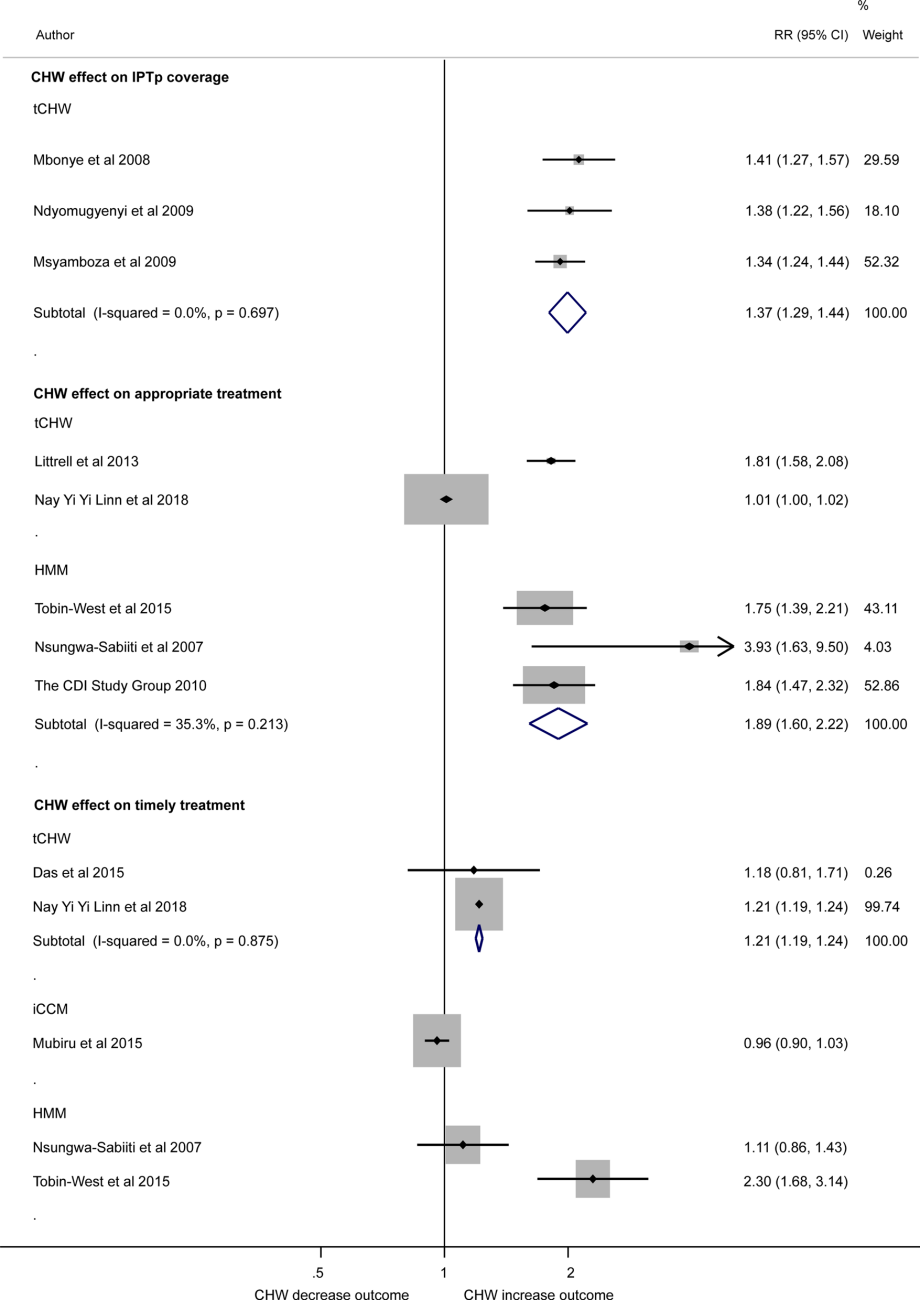
Forest plot showing inverse variance meta-analysis of the effect of CHW on IPT in pregnancy (2 doses) coverage; random effect analysis of the association between use of CHW, and appropriate and timely (treatment within 24 h after onset of fever) treatment for fever on under-5 children. Plot shows risk ratios (RR), 95% confidence intervals (95% CI) and inverse variance study weights (% weight). Estimates were calculated from data in the papers. Please note: due to high heterogeneity results were not pooled across studies quantifying the effect of community-delivered models on appropriate treatment for fever on under-5 children and timely treatment for fever on under-5 children (I-squared = 96.9% and = 93.3%, respectively)

Meta-analysis that pooled three studies using the tCHW model (Fig. 3), demonstrated a 37% increase in IPT coverage in pregnancy (IPTp) (pregnant women who completed two doses of sulfadoxine-pyrimethamine in the tCHW arm compared to the non-CHW arm (IPTp from other providers) (RR = 1.37 95% CI 1.29, 1.44; I-squared = 0.0%). One further study demonstrated that delivery of IPTp (defined as first dose of sulfadoxine-pyrimethamine in 2nd trimester) was 16.3% higher in the tCHW intervention arm compared to non-CHW arm (RR = 1.16 95% CI 1.15, 1.17) [32]. Two studies investigated the association between tCHW model and coverage of IPT in children (children who received all four courses of IPT) and both showed no association (RR = 1.03 (95% CI 0.92, 1.16) [33] and OR 1.18 (95% CI 0.62, 2.22) [34]).

#### Children who received appropriate or timely treatment for fever

Meta-analysis found high heterogeneity among the five studies using tCHW (n = 2) and HMM (n = 3) to assess appropriate treatment (Fig. 3). It demonstrated that the relative risk of receiving appropriate treatment of fever for under-5 children increased in intervention arm (CHW provided services) compared to the non-CHW arm (other providers provided services) in one tCHW study (RR = 1.81 95% CI 1.58, 2.08) [35] and pooled analysis of 3 HMM studies (RR = 1.89 95% CI 1.60, 2.22 (I-squared = 35.3%). However, no association was found in another tCHW study (RR = 1.01 (95% CI 1.00, 1.02) [36].

Meta-analysis also found high heterogeneity among five studies that assessed timely treatment (Fig. 3). The pooled analysis of two tCHW studies [26, 36] RR = 1.21 (95% CI 1.19, 1.24) and another study investigating HMM [31] RR = 2.30 (95% CI 1.68, 3.14) found that community-delivered models significantly increased the coverage of under-5 children receiving timely treatment for fever in CHW arm compared to non-CHW arm. However, one study investigating HMM [37] RR = 1.11 (95% CI 0.86, 1.43) and another one for iCCM [38] RR = 0.96 (95% CI 0.90, 1.03) had no difference in timely treatment for fever (under-5 children treated for fever within 24 h) in intervention arm compared to non-CHW arms.

### Impact of community-delivered models on malaria-metric indices

#### Parasitaemia and hyperparasitaemia

**Fig. 4:**
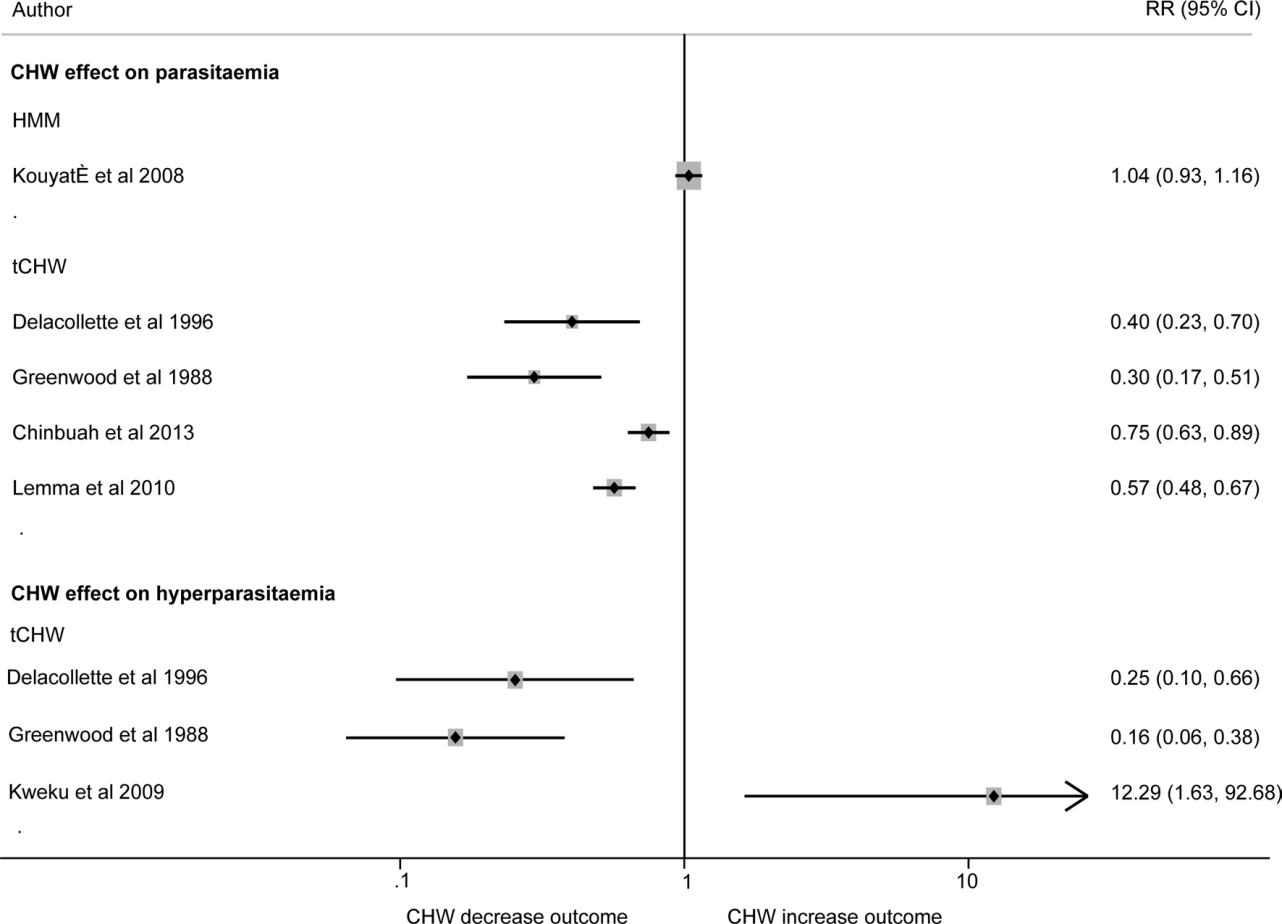
Random effect analysis of the association between use of CHW and impact on parasitaemia and hyperparasitaemia. Plot shows risk ratios (RR) and 95% confidence intervals (95% CI). Estimates were calculated from data in the papers. Parasitaemia was defined as presence of any malaria parasite species [40, 41, 43, 48], or *P. falciparum* [39] by microscopy. Hyperparasitaemia was defined as parasite density 7000/μl [34], parasitaemia 5000/μl and over [41] and more than or equal to 2000 asexual forms of *P. falciparum* per mm [40] in the blood detected by microscopy. Due to high heterogeneity results were not pooled across studies quantifying the effect of community-delivered models on parasitaemia or hyperparasitaemia (I-squared = 93.4% and 87.5% respectively)

While heterogeneity prevented pooled analysis of five studies that assessed parasitaemia (I-squared = 93.4%), all four studies that investigated the tCHW model showed reduced prevalence of parasitaemia (by 25–70%) compared to non-CHW arms, while one study that used HMM [39] demonstrated no difference in parasitaemia when malaria services were distributed by HMM or non- community-delivered models (RR = 1.04 95% CI 0.93, 1.16) (Fig. 4).

There was also high heterogeneity in the three tCHW studies reporting hyperparasitaemia (I-squared = 87.5%). tCHW provided services significantly reduced hyperparasitaemia (by 75% or more) in two studies compared to non-CHW arms, RR = 0.25 (95% CI 0.10, 0.66) [40] and RR = 0.16 (95% CI 0.06, 0.38) [41]. However, another study demonstrated increased risk of hyperparasitaemia in the tCHW group (RR = 12.29 95% CI 1.63, 92.68) [34] (Fig. 4).

#### Malaria cases

**Fig. 5:**
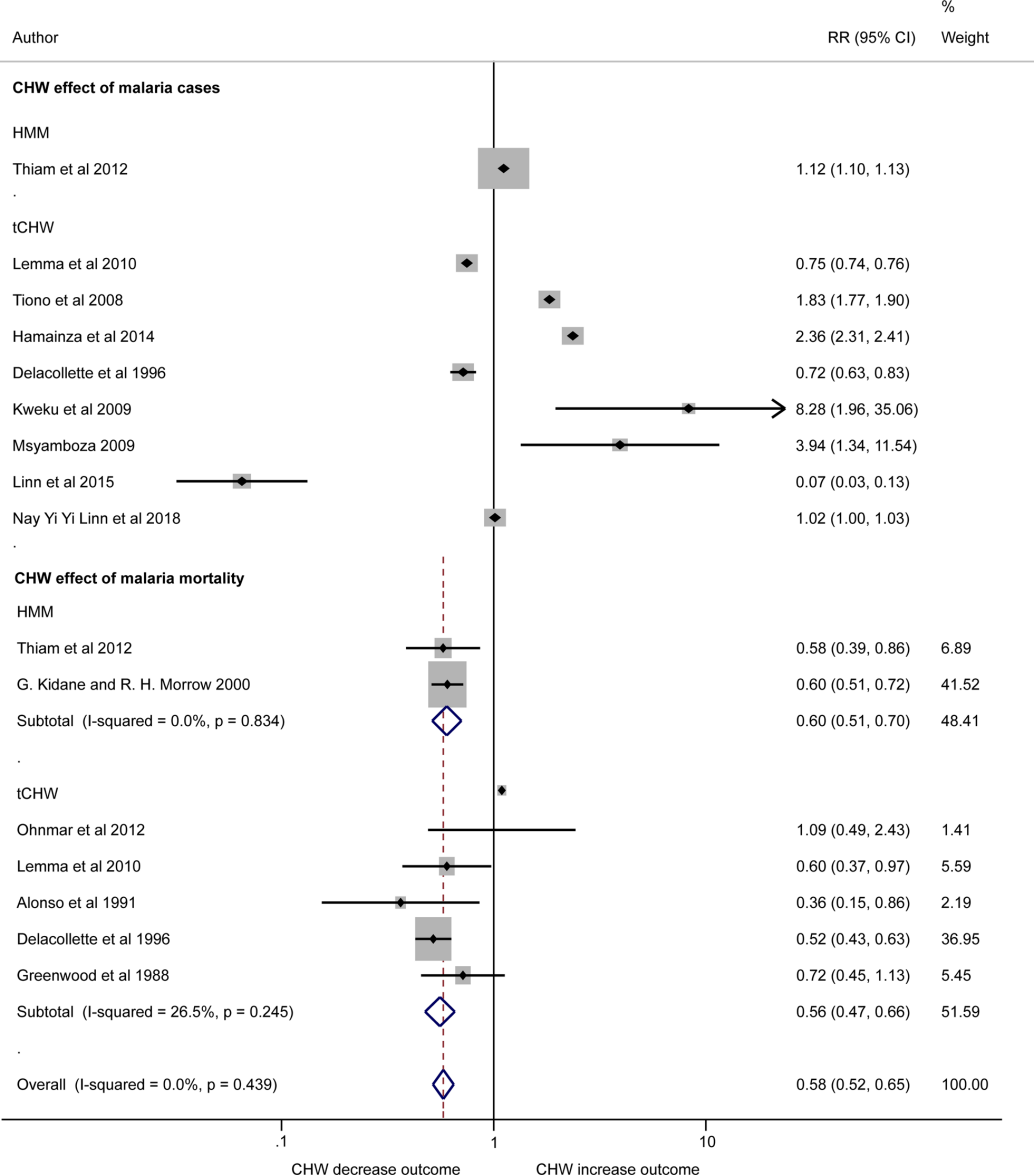
Forest plot showing the impact of CHW on malaria clinical cases and malaria mortality. Plot shows risk ratios (RR), 95% confidence intervals (95% CI) and inverse variance study weights (% weight). Estimates were calculated from data in the papers. Malaria cases are confirmed by parasitological test [34, 40, 42–44, 46, 47] and presumptive clinical diagnosis [45]. Please note: due to high heterogeneity results were not pooled across studies quantifying the effect of community-delivered models on clinical malaria cases (I-squared = 99.9%)

Nine studies compared the effect of tCHW (n = 8) and HMM (n = 1) on difference in malaria caseload in the intervention arms compared to non-CHW arms. Between-study heterogeneity was extremely high (I-squared = 99.9%) and HMM had negative effect (CHW arm had increased caseload) RR = 1.12 (95% CI 1.10, 1.13) [42]. tCHW revealed positive effect (CHW decrease malaria cases in the community) in three studies [40, 43, 44], negative results in four studies [34, 45–47] and no association in one study [36] (Fig. 5).

#### Malaria mortality

Both HMM and tCHW models in the meta-analysis (n = 7) showed that CHW intervention arms had lower malaria death rates compared to the non-CHW arms, with pooled RR = 0.58 (95% CI 0.52, 0.65) (I-squared = 0.0%) (Fig. 5).

#### Anaemia

**Fig. 6:**
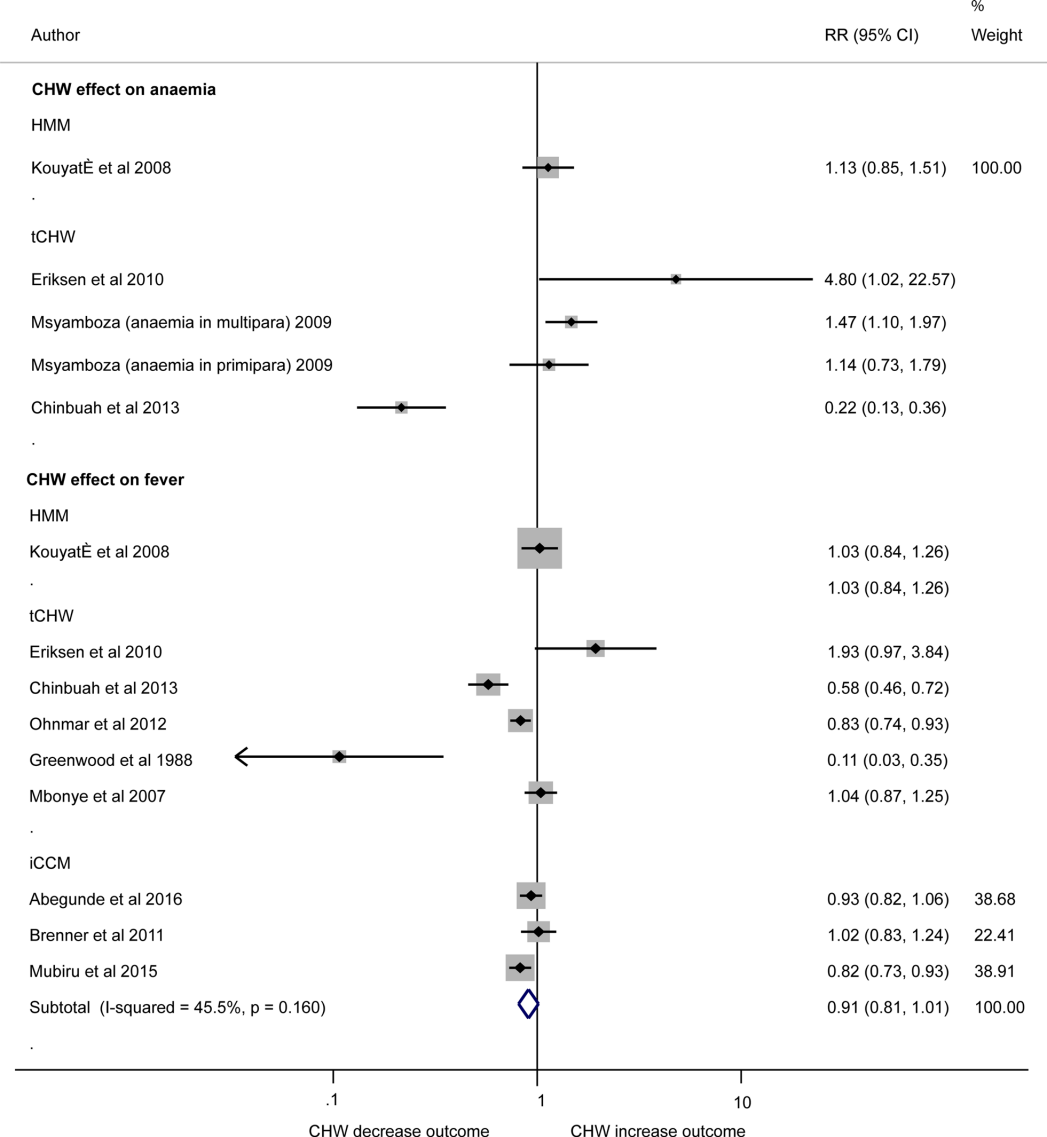
Forest plot showing the impact of CHW on anaemia incidence and fever prevalence by random effect analysis. Plot shows risk ratios (RR) and 95% confidence intervals (95% CI). Estimates were calculated from data in the papers. Definitions: Anaemia is defined as Hb < 8.0 g/dl [47–49] or haematocrit ≤ 24% [39]; Fever was defined as body temperature more than 37.5 °C [28, 30, 38, 39, 41, 48, 49] or reported fever cases [27, 56]. Studies investigating fever included women who had fever since delivery of last child [56]; fever plus parasitaemia [41], fever prevalence within last 1 month [30, 39, 48, 49] and last 2 weeks [27, 28] in general population; and fever prevalence [28] in under 5 children [38]. Please note: due to high heterogeneity results were not pooled across studies quantifying the effect of community-delivered models on risk of anaemia or fever (I-squared = 91.9% and 80.9%, respectively)

The four studies investigating the impact of the use of community-delivered model on the incidence of anaemia were methodologically diverse investigating specific high risk populations so meta-analyses were not performed (Fig. 6). One study showed that the relative risk of anaemia in under-5 children was 78% lower (RR = 0.22, 95% CI 0.13, 0.36) in the tCHW arm compared to non-CHW arm [48], conversely another tCHW study in the general population [49] found a Relative Risk (RR) = 4.8 times higher (95% CI 1.02, 22.57) and a tCHW study in multiparous pregnant women showed RR = 1.47 times higher (95% CI 1.10, 1.97) in CHW arm compared to non CHW arm (with no effect seen in primiparous women) [47]. One study showed no association between HMM and anaemia in general population (RR = 1.13 95% CI 0.85, 1.51) [39].

#### Fever

Meta-analysis showed considerable heterogeneity between nine studies that compared the impact of the use of community-delivered models on fever prevalence compared to non-CHW arms. Effects by different community-delivered models showed no difference in HMM (RR 1.03 95% CI 0.84, 1.26) [39], mixed results across five heterogeneous tCHW studies (two showed no effect, with RR 1.93 (95% CI 0.97, 3.84) [49] and 1.04 (95% CI 0.87, 1.25) [32], while three studies revealed significant reduction of fever cases, with RR 0.58 (95% CI 0.46, 0.72) [48], 0.83 (95% CI 0.74, 0.93) [30], and 0.11 (95% CI 0.03, 0.35) [41], respectively), and mixed results for three iCCM studies (two studies found no association (RR 0.93 95% CI 0.82, 1.06) [27] and 1.02 (95% CI 0.83, 1.24) [28]) while one study showed positive effect, RR = 0.82 (95% CI 0.73, 0.93) [38] (Fig. 6).

## Discussion

This review aimed to provide a holistic review of the impact of community-delivered models on all available outcome indicators for malaria intervention coverage and their impact on malaria metric indices. Implementation of community-delivered models (which included CHW, HMM and iCCM) typically increased coverage of malaria interventions (such as bed nets and IPTp) and reduced the risk of parasitaemia and malaria-attributed mortality, but not proxy measures of malaria (such as fever and anaemia) compared to malaria interventions delivered by non-community-delivered models. There was significant methodological and statistical heterogeneity and variations in internal validity, of the included studies, which made meta-analysis and formal comparisons of the impact of different community-delivered models difficult. The majority of studies were conducted in high transmission settings in Africa, and focused on high risk sub-groups such as pregnant women and under-5 children, which has implications on the generalizability of findings to low transmission and elimination settings, such as areas in Asia, where the epidemiology of malaria is different.

Community-delivered models have been widely used to distribute bed nets and implement appropriate malaria diagnosis and treatment in the community. This review found that community-delivered models increased bed net coverage up to 54%. The included papers attributed these improvements to the fact that CHW were local peer providers, received proper training and intensive supervision and were tasked with helping with the community mobilization and logistical aspects [28] and that CHW reduced the additional hindrances to accessing appropriate services in the field [50]. However, while large magnitudes of effect were observed for bed net coverage, pooled analysis demonstrated that community-delivered models only increased bed net usage by 4% compared to those delivered by non-community-delivered models. Encouraging and promoting proper use of malaria interventions, such as bed nets, over and above ensuring physical access to such interventions, should be a key component of community-delivered models to improve the use of malaria services in the community. This review also identified studies providing evidence for the delivery of less routinely delivered malaria interventions through community-delivered models. IPTp is typically distributed to pregnant women attending antenatal clinics and distribution through tCHW increased IPTp (2 dose) coverage (37%) compared to antenatal clinics. This highlights that IPTp can be distributed successfully through tCHW and could fulfil service gaps in countries where IPTp is policy. Overall, this review provides evidence for the advantage of community-delivered models over non-community-delivered models for malaria control and possible expansion of malaria services through community channels to supplement facility based services.

Implementation of community-delivered models also had a positive impact on parasitaemia and malaria-attributed mortality, but not proxy malaria metric measures, such as anaemia and fever. Community-delivered models reduced the risk of parasitaemia (from 25 to 70%, for tCHW only) and malaria-attributed mortality (by 42% in pooled analysis) compared to non-community- delivered models. All tCHW studies [40, 41, 43, 48] that demonstrated a significant reduction of parasitaemia in the community specified the use of a simple treatment algorithm (adapted from a national/international guideline) to be followed by CHWs. In the studies that demonstrated a reduction of malaria-attributed mortality, CHW provided malaria diagnosis, appropriate treatments and referral services according to the national guidelines (but no simplified algorithm specified). Therefore, the national malaria management guideline of an endemic country should be simplified and CHWs supported so that community-delivered models yield direct impacts on malaria-metrics indices.

The positive impact of community-delivered models on increasing malaria services coverage, and reducing parasitaemia and malaria mortality were consistent despite methodological heterogeneity between studies, which strengthens the evidence for the use of community-delivered models to deliver malaria services. However, heterogeneity observed across studies that evaluated proxy indicators of malaria (anaemia and fever) contributed to the lack of clear overall effect. This was most likely due to methodological heterogeneity in the community models utilized (tCHW, iCCM and HMM), to differences in the malaria interventions delivered in community-delivered models, and in the comparison groups used, all of which made the studies unalike and difficult to compare. Interventions delivered by CHW for studies evaluating the impact on anaemia and fever were particularly diverse. For example, one study [49] only provided IPTp and behavioural change communication while others provided comprehensive malaria services, such as bed net distribution, parasitological diagnosis, treatment and referral of malaria cases [46]. In addition to heterogeneity, evaluation of bias revealed that the included studies were moderately to seriously biased, which would have impacted on the internal validity of the findings as well as strength and directions of estimates. Other sources of bias should also be considered. While publication bias could not be assessed adequately in this review due to the small number of studies in each analysis, publication bias may still have affected the number of studies included as well as the direction of estimates of effect. However, this bias was mitigated by including searches of the grey literature. Measurement of the aetiologies of anaemia and fever are multifactorial and the estimates were subject to information bias (for example, in the case of self-reported fever). The methodological heterogeneity and bias of included studies may affect the strength of the evidence of the use of community-delivered models for malaria control and elimination.

This review aimed to be as inclusive as possible and included all relevant and available studies regardless of language and dates of publication; previous published reviews, published up until 2014, were restricted to particular models, interventions, target populations or geographical areas and only a review included meta-analyses [17]. This holistic systematic review enabled comparisons and quantification of heterogeneity across the aforementioned factors and performed meta-analyses including several more recently published studies. Compared to previous review findings the magnitudes of effect in pooled analyses of CHW on increasing coverage of bed nets [11, 17] and IPTp [11] and reducing the risk of parasitaemia [10, 11, 17]  and malaria attributed mortality [10, 15] were generally lower in this review as previous meta-analyses only included studies conducted in high transmission areas of sub-Saharan Africa and focused on high risk population groups.

However there were limitations in the range of geographical and malaria transmission settings, influence of factors independent of community-delivered models and integration of health services which may impact the generalizability of the findings in different contexts. Most studies were conducted in Africa and were undertaken in malaria control settings, with control-specific indicators such as malaria interventions coverage and disease burden. Elimination-specific indicators such as number of foci by classification, number of people and percentage of population living in active foci, and number of malaria deaths by species and by imported or locally acquired [51] were not included as none of the identified studies were undertaken in elimination setting. This gap highlights that the magnitude of effects of the community-delivered models on coverage and malaria-metric indices might not be the same in the elimination settings of low transmission areas compared to control settings in the African Region.

Furthermore, the success of the community-delivered models in malaria control and elimination relies on implementation of the model such as assignment, recruitment, training, supportive supervision and ongoing technical support to CHWs, incentive for CHWs, and management structure in a ministry or an organization that manages CHW program which is nested in the broader and complex national health system. The generalizability of the findings from this review depends on health system factors independent of community-delivered models, such as budget allocation, policy of intervention distribution in the respective country and effectiveness of the logistic and supply chain systems [52, 53]. Therefore, caution is needed in translating the effectiveness of community-delivered models in different context-specific malaria programmes. Moreover, the community-delivered models identified in this review have limitations in the integration of services for common diseases in the community other than malaria. Only the iCCM model integrated services for common childhood illnesses; tCHW and HMM were primarily for malaria alone. Hence, the effectiveness of community-delivered models may vary depending on what other health interventions are included in the integrated community models. No integrated model combines interventions that cover both adult and child high-burden diseases in the global burden list; implementation and evaluation of such models in the malaria elimination setting represent a global research gap.

## Recommendations and conclusions

Overall, the community-delivered model is an effective approach to control malaria in high transmission areas. However, community-delivered models need to be supported by a number of independent systematic factors such as country specific health system to make the model most effective in different country contexts. Currently, endemic countries use integrated community- delivered models although the knowledge on what health problems to be integrated optimally into the community-delivered model of each endemic country remains a gap. Moreover, elimination program-specific community-delivered models are yet to be implemented and evaluated as suggested in malERA: An updated research agenda for health systems and policy research in malaria elimination and eradication [54]. To maintain the effectiveness in elimination programs in the context of primary health care, community-delivered models need to be supported, advanced and adapted throughout the implementation of CHW recruitment, training, supportive supervision and ongoing technical support, and provision of incentives to CHWs, considering the changing malaria epidemiology, development of new tools and technologies, and dynamic political environment of each malaria endemic country. Further country-specific series of operational research for development of community-delivered integrated malaria elimination model are essential in order to provide an evidence base for the integration of interventions for different diseases and to maintain the effectiveness of community-delivered model in national malaria elimination programme.

## Additional files

**Additional file 1.** Different names of community health workers (CHW).

**Additional file 2.** The protocol for this systematic review.

**Additional file 3.** Search strategy, syntax and source of grey literature searches.

**Additional file 4.** Papers excluded after second step screening with reasons for exclusion.

**Additional file 5.** Quality assessment by ‘Risk Of Bias in Non-randomized Studies—of Interventions (ROBIN-I)’ for Non-randomized studies and by ‘Cochrane Collaboration’s Tool for assessing risk of bias’ for Randomized studies.

## Data Availability

All data generated or analysed during this study are included in this published article and its additional files.

## Abbreviations

ACT: artemisinin-based combination therapy
BCC: behavioural change communication
CHW: community health workers
HMM: home management of malaria
iCCM: integrated community case management
IPT: intermittent preventive treatment
IPTc: intermittent preventive treatment in children
IPTp: intermittent preventive treatment in pregnancy
ITN: insecticide treated net
LILACS: Latin American and Caribbean Health Sciences Literature
LLIN: long-lasting insecticidal net
M: malaria interventions
M+: malaria plus other diseases interventions
MOOSE: meta-analysis of observational studies in epidemiology
PRISMA: preferred reporting items for systematic reviews and meta-analyses
PROSPERO: international prospective register of systematic reviews
RDT: rapid diagnostic test
ROBINS-I: the risk of bias in non-randomized studies-of interventions
RR: relative risk
tCHW: traditional CHW
WHO: World Health Organization
