# Supplementary material for: The impact of community-delivered models of malaria control and elimination: a systematic review
Source: Malar J. 2019 Aug 6;18:269. doi: 10.1186/s12936-019-2900-1 (PMC6683427; doi:10.1186/s12936-019-2900-1)
Supplement: Supplementary file 1 — Additional file 1. Different names of community health workers (CHW). [file 12936_2019_2900_MOESM1_ESM.docx]

**Additional material 1: different names of community health workers (CHW)**

Malaria Control Assistances (MCAs), Community Medicine Distributors (CMDs), Traditional Birth Attendants (TBAs), Community Reproductive Health Workers (CRHWs), Drug-shop Vendors (DSVs), Adolescent Peer Mobilisers (APMs), Community Health Workers (CHWs), Community-directed Distributors (CDDs), Drug Distributers (DDs), Health Extension Workers (HEWs), Village Malaria Workers (VMWs), Village Health Workers (VHWs), Women Group Leaders (WGL), Home Care Providers (HCPs), Patent Medicine Vendors (PMVs), chemists, Accredited Social Health Activist (ASHAs), community volunteers, Mobile Malaria Workers (MMWs), Community Health Post (CHPs), Malaria Microscopists (MMs), Hamlet Health Workers (HHWs), women leaders, mother coordinators, Community Owned Resource Persons (CORPs), Volunteer Home Care Provider (HCPs), Key Opinion Leaders (KOLs), mother trainers, Community Oriented Resource Persons (CORPs), Village Health Team Members (VHTs), Volunteer Community Health Workers (VCHWs), Agente Polivalente Elementares (APEs) are the different names of the CHWs used in the included papers that reflect their roles in the community.

In this review, the CHW includes grass root level staff either fully or partially employed by government health system or other health organizations however seamlessly integrated in the community (e.g. volunteer, service provider, staff, community worker, basic health worker, primary health care provider, traditional birth attendance, Auxiliary Midwife (AMW), Public Health Supervisor (PHS)).
