# Supplementary material for: The impact of community-delivered models of malaria control and elimination: a systematic review
Source: Malar J. 2019 Aug 6;18:269. doi: 10.1186/s12936-019-2900-1 (PMC6683427; doi:10.1186/s12936-019-2900-1)
Supplement: Supplementary file 2 — Additional file 2. The protocol for this systematic review. [file 12936_2019_2900_MOESM2_ESM.docx]

**Additional material 2: the systematic review protocol**

**Title: Community-delivered models of malaria control and elimination: Protocol for a systematic review**

**Administrative Information**

**Registration**:

In accordance with the guidelines, our systematic review protocol was registered with the International Prospective Register of Systematic Reviews (PROSPERO) on 14.12.2016 (registration number - CRD42016052929).

**Authors:**

1. Win Han Oo
   1. PhD student, School of Health and Social Development, Faculty of Health, Deakin University, [owinhan@deakin.edu.au](mailto:owinhan@deakin.edu.au)
   2. Program Manager, Burnet Institute Myanmar, winhanoo@burnetmyanmar.org
2. Freya Fowkes
   1. Associate Professor, Head of Malaria and Infectious Diseases Epidemiology Group, Burnet Institute, freya.fowkes@burnet.edu.au
3. Lisa Gold
   1. Associate Professor, School of Health and Social Development, Faculty of Health, Deakin University, lisa.gold@deakin.edu.au
4. Kerryn Moore
   1. PhD candidate, University of Melbourne, [kerrynmoore.kam@burnet.edu.au](mailto:kerrynmoore.kam@burnet.edu.au)

Contact person (corresponding author):

Win Han Oo

226,4^th^ Floor, U Wisara Road, Wizaya Plaza, Bahan Township, 11201, Yangon

Ph: +95 1 375785 (Ext 307), Mobile: +95 99 7490 2209

Email: [owinhan@deakin.edu.au](mailto:owinhan@deakin.edu.au); [winhanoo@burnetmyanmar.org](mailto:winhanoo@burnetmyanmar.org)

Dates: Assessed as up to date - 6.12.2016

Date of search - September 2016

Protocol first published - December 2016

Review first published -

Last citation issue -

Contributions:

Win Han Oo is the guarantor. Freya Fowkes and Lisa Gold provide technical supervision on all aspects. Kerryn Moore is an independent data extractor as well as quality assessor of the included papers that will lead to synthesis and write up.

**Support:**

This systematic review is funded and supported by the Deakin University and Burnet Institute through the Deakin-Burnet PhD scholarship. School of Health and Social Development, Deakin University and Burnet Institute have overall control of the data. The funder will have no input on the interpretation or publication of the study results.

**Introduction**

Malaria caused by *Plasmodium* spp. is an infectious disease transmitted by Anopheles mosquitoes. An estimated 3.2 billion people in the world are at risk of malaria and the WHO estimates that in 2015, malaria incidence was 214 million (range: 149–303 millions) cases and mortality due to malaria was 438 000 (range: 236 000–635 000) globally ([WHO, 2015b](#_ENREF_13)). The malaria burden has declined significantly since 2000 with a drop in malaria incidence and mortality rates of 30% and 48% respectively largely due to increased malaria control measures. An increasing number of countries are moving towards malaria elimination, whereas in 2015, 16 new countries declared malaria has been eliminated ([WHO, 2015b](#_ENREF_13)).

A numbers of key interventions have been used to control malaria targeting the agent, host and vector. These include vector control, personal protection (LLIN/ITN interventions), chemoprevention for malaria prevention, and early diagnosis and appropriate treatment usually through Passive Case Detection (PCD) at public and private health facilities, community based diagnostic testing and effective treatment, integrated community based diagnostic testing and treatment, screening and treatment at malaria posts or universal access to quality diagnostic and treatment facilities for case management. Important intervention includes Behavioral Change Communications (BCC) comprised of community based BCC activities, integrated community based health education, health facility based education activities. Market interventions to reducing counterfeit drug supply, increasing quality assured anti-malaria drugs are also used to control malaria. Recording, reporting and surveillance are crosscutting themes executed through community based surveillance, real time reporting from the community, health facility-based recording, reporting and surveillance. Basic science and operational research for malaria also play a crucial role for malaria control.

WHO suggests that all countries suffering from malaria should accelerate efforts towards malaria elimination through combinations of interventions tailored to local contexts. Innovative tools and implementation approaches will be required to enable countries to achieve their progression along the path to elimination ([WHO, 2015a](#_ENREF_12)). Targeting malaria elimination needs more aggressive interventions addressing the infection and parasite rather than just intensifying the control phase interventions. Potential additional interventions include Active Case Detection (ACD), mass screening and treatment, scaling up of elimination standard community case management, Directly Observed Treatment (DOT) and follow up of each and every malaria case, identification of pocket area, classification and appropriate management, notification of malaria cases and case investigation and classification, contact tracing and delivery of appropriate interventions, universal coverage of Long Lasting Insecticidal Nets (LLIN) and expansion of other personal protective measures, setting up elimination standard recording and reporting system down to the community ([WHO, 2015a](#_ENREF_12)).

A key challenge in malaria control is delivery of malaria interventions to the communities that need them most. Different models have been used to reach to the beneficiaries and include:

Government health facility based models in that most of the interventions are distributed through government health facilities and programs,

Non-government not-for-profit health facility based models which provide the measures via non-government not-for-profit health facilities and clinics,

For profit health facility based model in which the services are delivered through private sector, and Community-delivered models in which the interventions are delivered via Community Health Workers (CHW) and rely on community participation and engagement.

The use of community-based delivery methods which can be delivered are increasing. They can be implemented with minimal training ([WHO, 2015b](#_ENREF_13)), are effective and cost-effective in resource limited countries and several health areas ([McCord, Liu, & Singh, 2013](#_ENREF_5); [Rasanathan et al., 2014](#_ENREF_6); [The_CDI_Study_Group, 2009](#_ENREF_9)). Different community-delivered models for malaria control and elimination have been developed and implemented either as malaria interventions alone or as an integrated program together with interventions for other diseases and health problems. In a community-delivered model, community health workers are the key players and the desired interventions are delivered through them.

WHO defines Community-health workers (CHW) as “*Community health workers should be members of the communities where they work, should be selected by the communities, should be answerable to the communities for their activities, should be supported by the health system but not necessarily a part of its organization, and have shorter training than professional workers” (*[*WHO, 2007*](#_ENREF_11)*)P1.*

In a numbers of settings, community health workers/volunteers are assigned for malaria and childhood infectious diseases mainly for pneumonia and diarrhea ([Smith Paintain et al., 2014](#_ENREF_7); [Velema, Alihonou, Gandaho, & Hounye, 1991](#_ENREF_10)). Their quality of performance has been questioned in some settings ([Yansaneh et al., 2016](#_ENREF_14); [Yasuoka et al., 2010](#_ENREF_15)) and the success of these models has varied. Further investigation is needed of the different types of community-delivered interventions and models (malaria alone or malaria plus other diseases models) and their relative effectiveness in increasing coverage and decreasing malaria-metric indices, as well as barriers and enablers that facilitate the development of feasible, context-specific malaria elimination model(s) that could be used for the successful control and elimination of malaria in different settings.

**Aim:**

To investigate the impact of community-delivered malaria intervention programs and barriers and enablers of successful implementation

**Review Questions:**

1. What are the community-delivered models which have been used to deliver malaria or malaria plus other diseases interventions to individuals living in areas at risk of malaria?
2. What change in coverage in malaria or malaria plus other diseases interventions to individuals living in areas at risk of malaria is achieved with community-delivered models?
3. What is the impact of community-delivered models on malaria-metric indices?
4. What are the barriers and enablers of the identified community delivered models?

**Objectives:**

1. To identify community-delivered models for malaria or malaria plus other diseases interventions
2. To quantify the impact of community-delivered models on coverage of malaria or malaria plus other diseases interventions
3. To quantify the impact of community-delivered models on malaria-metric indices
4. To identify the barriers and enablers of the identified community-delivered models

**Methods**

The study is a systematic review of published and grey literature of quantitative and qualitative studies investigating community- delivered models for delivering malaria or malaria plus other diseases interventions for malaria control and elimination.

**Types of studies**

All study designs are included (observational studies: cross-sectional, case-control and cohort studies; and experimental studies: observational intervention studies, controlled trials and quasi-experimental studies) that contain quantitative or qualitative data describing the outcomes or processes involved in community-based delivery of malaria or malaria plus other diseases interventions. Grey literature that addresses the objectives of the review will be included. If existing systematic reviews with similar research questions and objectives are identified, these will be discussed first in the report.

Inclusion criteria:

- Studies used or addressed community based model in delivery of malaria or malaria plus other diseases interventions

- Impact(s) were measured in terms of coverage, malaria-metric indices and/or barriers and enablers in distribution

Exclusion criteria:

- Studies that do not use community delivered models.

- Papers that do not present new data or unable to retrieve new data (case series, opinion pieces, reviews, editorials, conference abstracts, study protocols, posters),

- Existing systematic reviews with similar research questions and objectives,

- Studies or interventions that do not involve malaria

- Studies or interventions that do not address the review objectives

- Studies or interventions that are not for disease(s) control or elimination

- The population studied is not the eligible population described in the review protocol

**Types of participants**

The population to be studied is individuals living in areas at risk of malaria (stable areas where Plasmodium Falciparum Annual Parasite Index (PfAPI) ≥0.1 per 1,000 pa & unstable areas where PfAPI > 0 and < 0.1 per 1,000 pa) as defined by the Malaria Atlas Project ([Gething et al., 2011](#_ENREF_3)) who have access to community-delivered models of malaria control, and CHWs providing malaria interventions to their community. Health care and associated staff who have direct or indirect interactions with participants in the community-delivered models.

**Types of interventions**

All malaria interventions distributed in community-delivered models through CHWs will be included.

For the purpose of this systematic review the CHW will include grass root level staff either fully or partially employed by government health system or other health organizations however seamlessly integrated in the community (eg volunteer, service provider, staff, community worker, basic health worker, primary health care provider, traditional birth attendance, Auxiliary Midwife (AMW), Public Health Supervisor (PHS)).

**Search methods for identification of studies**

Searches will include peer-reviewed publications and grey literature available online. Data sources will include journal publications, evaluation reports, policy guidelines and strategy documents. The reference lists of the included papers will also be reviewed. The first author (WHO) will independently select studies for inclusion in two stages with inputs from FF & LG. Details are available in appendix 1 and 2. Firstly by screening of title and abstracts (peer reviewed papers) and title, list of content (grey literatures) against inclusion and exclusion criteria to identify potentially relevant studies then screening of full texts for final inclusion. Study selection will be carefully documented and reported. A list of excluded studies in second stage will also be reported, including reasons for exclusion for the papers excluded in second stage. References will be managed using EndNote reference manager software.

1) PubMed, 2) Embase, 3) Cochrane Central Register of Controlled Trials, 4) LILACS, 5) African Medicus Index will be searched with no restriction on publication date and language (although English will be used in data extraction)

A Boolean search query will comprise of:

(malaria OR plasmodium OR falciparum OR vivax)

“AND”

(health volunteer OR service provider OR staff OR community health worker OR basic health worker OR primary health care provider OR traditional birth attendan* OR auxiliary midwi* OR public health supervisor OR community intervention OR community model)

Additional searches will be made of grey literature databases with no restriction on publication date and language, focusing on those held by agencies working on malaria. These include:

- UN agencies (World Health Organization <http://www.who.int/en/> , UNICEF <http://www.unicef.org/> , UNOPS <https://www.unops.org/english/Pages/Home.aspx> );
- International Organizations (IO) and Non-Government Organizations (NGO) (Population Services International <http://www.psi.org/> , Malaria Consortium <http://www.malariaconsortium.org/>
- International Organizations for Migration <http://www.iom.int/> , Save the Children International <https://www.savethechildren.net/> , Médecins Sans Frontières (MSF) International <http://www.msf.org/> , Community Partners International <http://cpintl.org/> );
- Philanthropies and donor agencies (Bill and Malinda Gate Foundation <http://www.gatesfoundation.org/> , United States Agency for International Development <https://www.usaid.gov/> , UK Department for International Development <https://www.gov.uk/government/organisations/department-for-international-development> , Australian Department of Foreign Affairs and Trade <http://dfat.gov.au/pages/default.aspx> , Asia Development Bank <http://www.adb.org/> , Japan International Cooperation Agency <http://www.jica.go.jp/english/> ).

**Study Records**

**Outcome measures**

**Objective 1:**

- Identification of range of community-delivered malaria or malaria plus other diseases intervention models
- Identification of range of malaria interventions delivered by each community-delivered model

**Objective 2:**

- Coverage (number and/or percentage, or other measures of service uptake) of malaria or other diseases interventions delivered by community-delivered models

**Objective 3:**

- Number and/or percentage of people diagnosed with malaria by microscopy, Rapid Diagnostic Test (RDT) or Polymerase Chain Reaction (PCR)
- Number and/or percentage of malaria cases
- Number and/or percentage of people died from malaria

**Objective 4:**

- Quantitative and qualitative measures of barriers and enablers of the community-delivered malaria intervention models

1. **Data collection and analysis**

**Data extraction**: W H Oo and K Moore will independently undertake data extraction using data extraction proforma in Appendix 3. Discrepancies will be resolved with discussion with FF and LG. If further information is required that is not featured in the literature, the authors will be contacted up to two times via email.

The data extraction structure has been informed by the SURE Collaboration’s guides to policy-relevant barriers and enablers (<http://global.evipnet.org/SURE-Guides/>). Data extraction will cover the following categories:

- Basic study information, type of community- delivered model, participants and malaria interventions and additional service/s
- Integration intervention, service delivery option/s, nature of models
- Outcomes including community models and interventions, coverage, achievement by indicators, other uptake measure and enablers and barriers of the models.

**Quality assessment** will be conducted with data extraction. A Cochrane Risk of Bias Assessment Tool: for Non-randomized Studies of Interventions (ACROBAT-NRSI) also known as The Risk Of Bias In Non-randomized Studies – of Interventions (ROBINS-I) assessment tool will be used for quantitative studies to assess the bias of the included studies, classifying studies in five levels: low risk, moderate risk, serious risk and critical risks of bias (in ascending order of risk), and no information.

ROBINS-I assessment is based on the seven domains of bias: bias due to confounding, bias in selection of participants into the study, bias in classification of interventions specified for pre-intervention; bias due to deviations from intended interventions specified for at-intervention, bias due to missing data, bias in measurement of outcomes, and bias in selection of the reported result specified for post-intervention phases (the tool and detail explanations are available at <https://sites.google.com/site/riskofbiastool/> ).

A checklist derived from the Critical Appraisal Skills Program (CASP) ([Critical Appraisal Skills Programme (CASP), 2014](#_ENREF_2)) will be used for quality assessment of qualitative data and qualitative studies <http://media.wix.com/ugd/dded87_29c5b002d99342f788c6ac670e49f274.pdf> .

The quality assessment will be independently completed for each study by two review authors (W H Oo and K Moore).

**Data**

**Synthesis of findings**:

Information from the included papers will be extracted and classified into qualitative and quantitative data. Qualitative data will be summarized as a comparative content analysis facilitated by matrix displays. Quantitative data will be summarized as a descriptive analysis (and inferential analysis whenever possible) using frequencies, distributions, ratios, means and proportions. Very low quality documents (those rated as serious risk and critical risk of bias) will result in exclusion of studies from impact indicators analysis, effectiveness analyses, and/or quantitative meta-analysis.

Different types of community based models will be identified based on the findings and the studies and intervention programs will be categorized accordingly.

Meta-analysis will be attempted where community models prove to be sufficiently alike in setting and nature but comparable. This will examine relative and absolute differences in models between intervention populations and comparator populations and may need to group meta-analyses by study type: control vs. intervention or pre-/post-intervention.

Dichotomous data (coverage in terms of service uptake and/or geographical coverage, quantitative measures of barriers and enablers) will be determined by using proportions with 95% confidence interval (CI). Continuous outcomes (number and/or percentage in coverage, number and/or percentage of people diagnosed with malaria by RDT, microscopy or PCR, number and/or percentage of people died from malaria, quantitative measures of barriers and enablers) will be analyzed using difference in means (with 95% CI) or if the sample size is too low, difference in medians will be measured. Skewed data will be presented descriptively. STATA 13 will be used for the analysis. The coverage and impact indicators will be presented in forest plot whenever the included studies use the same coverage and impact indicators and are comparable in settings.

If the studies are too heterogeneous or if quantitative synthesis is not appropriate, a narrative synthesis using conceptual frameworks will be undertaken to identify common themes across included studies. The study designs and characteristics of the included studies will also be analyzed. We will try to explain the source of heterogeneity by subgroup analysis (based on the type of community model and/or intervention) or sensitivity analysis (quality components, including full-text publications versus abstracts, preliminary results versus mature results, published versus unpublished data).

Narrative reporting of qualitative data synthesis will be structured around SURE guideline categories <http://global.evipnet.org/SURE-Guides/> ([SURE Collaboration, 2011](#_ENREF_8)). Detailed reporting structure is available in appendix 4.

**Meta-bias(es) and Confidence in cumulative evidence**

We will attempt to report our level of confidence in synthesis recommendations relating to quantitative measures by adopting GRADE for effectiveness studies ([Balshem et al.](#_ENREF_1)). As implemented in SURE Collaboration guidelines ([SURE Collaboration, 2011](#_ENREF_8)) this will provide some narrative indication of our confidence in effectiveness conclusions (such that they can be readily adapted into guideline discussions) across the domains of study limitations, inconsistent results across studies, indirectness of the evidence, imprecision, and publication bias. The potential for reporting bias will be further explored by funnel plots if ≥10 studies are available.

Similarly, the updated CerQUAL ([Lewin et al., 2015](#_ENREF_4)) framework will be used to score confidence in qualitative implementation considerations (barriers and enablers) across methodological limitations, relevance, coherence across studies, and adequacy of data.

**Study limitations**

Specific limitations in this review could be: (a) identification of grey literature; and (b) quality of included studies.

**Other procedure**

In general, the review will attempt to follow Cochrane Handbook for Systematic Reviews of Interventions available at <http://handbook.cochrane.org/v5.0.2/>

**Conflict of Interest**

Nothing to declare

**References**

Balshem, Howard, Helfand, Mark, Schünemann, Holger J., Oxman, Andrew D., Kunz, Regina, Brozek, Jan, . . . Guyatt, Gordon H. GRADE guidelines: 3. Rating the quality of evidence. *Journal of Clinical Epidemiology, 64*(4), 401-406. doi: 10.1016/j.jclinepi.2010.07.015

Critical Appraisal Skills Programme (CASP). (2014). CASP Qualitative Checklist <http://www.casp-uk.net/#!checklists/cb36> (Creative Commons License). from Critical Appraisal Skills Programme <http://www.casp-uk.net/#!checklists/cb36>

Gething, Peter W., Patil, Anand P., Smith, David L., Guerra, Carlos A., Elyazar, Iqbal RF, Johnston, Geoffrey L., . . . Hay, Simon I. (2011). A new world malaria map: Plasmodium falciparum endemicity in 2010. *Malaria Journal, 10*(1), 1-16. doi: 10.1186/1475-2875-10-378

Lewin, Simon, Glenton, Claire, Munthe-Kaas, Heather, Carlsen, Benedicte, Colvin, Christopher J., Gülmezoglu, Metin, . . . Rashidian, Arash. (2015). Using Qualitative Evidence in Decision Making for Health and Social Interventions: An Approach to Assess Confidence in Findings from Qualitative Evidence Syntheses (GRADE-CERQual). *PLoS Med, 12*(10), e1001895. doi: 10.1371/journal.pmed.1001895

McCord, G. C., Liu, A., & Singh, P. (2013). Deployment of community health workers across rural sub-Saharan Africa: financial considerations and operational assumptions. *Bull World Health Organ, 91*(4), 244-253B. doi: 10.2471/blt.12.109660

Rasanathan, K., Muniz, M., Bakshi, S., Kumar, M., Solano, A., Kariuki, W., . . . Diaz, T. (2014). Community case management of childhood illness in sub-Saharan Africa - findings from a cross-sectional survey on policy and implementation. *J Glob Health, 4*(2), 020401. doi: 10.7189/jogh.04.020401

Smith Paintain, L., Willey, B., Kedenge, S., Sharkey, A., Kim, J., Buj, V., . . . Ngongo, N. (2014). Community health workers and stand-alone or integrated case management of malaria: a systematic literature review. *Am J Trop Med Hyg, 91*(3), 461-470. doi: 10.4269/ajtmh.14-0094

SURE Collaboration. (2011). SURE Guides for Preparing and Using Evidence-Based Policy Briefs. <http://global.evipnet.org/SURE-Guides/>

The_CDI_Study_Group. (2009). Community-directed interventions for priority health problems in Africa: results of a multicountry study. *Bulletin of the World Health Organization (BLT) 88*(Past issues ), 481-560. doi: 10.2471/BLT.09.069203

Velema, J. P., Alihonou, E. M., Gandaho, T., & Hounye, F. H. (1991). Childhood mortality among users and non-users of primary health care in a rural west African community. *Int J Epidemiol, 20*(2), 474-479.

WHO. (2007). Community health workers: What do we know about them? , 6. Retrieved from WHO Health Workforce website: <http://www.who.int/hrh/documents/community_health_workers_brief.pdf>

WHO. (2015a). *Global technical strategy for malaria 2016-2030* W. H. Organization (Ed.) (pp. 29). Retrieved from World Health Organization database Retrieved from <http://www.who.int/malaria/areas/global_technical_strategy/en/> doi:<http://www.who.int/malaria/areas/global_technical_strategy/en/>

WHO. (2015b). World Malaria Report 2015 (Malaria, Trans.) (World Health Organization ed., pp. 280 (217)). Geneva, Switzerland World Health Organization

Yansaneh, A. I., George, A. S., Sharkey, A., Brieger, W. R., Moulton, L. H., Yumkella, F., . . . Diaz, T. (2016). Determinants of Utilization and Community Experiences with Community Health Volunteers for Treatment of Childhood Illnesses in Rural Sierra Leone. *J Community Health, 41*(2), 376-386. doi: 10.1007/s10900-015-0107-0

Yasuoka, J., Poudel, K. C., Poudel-Tandukar, K., Nguon, C., Ly, P., Socheat, D., & Jimba, M. (2010). Assessing the quality of service of village malaria workers to strengthen community-based malaria control in Cambodia. *Malar J, 9*, 109. doi: 10.1186/1475-2875-9-109

**Appendix 1 – Search strategy and syntax for 5 databases**

1. **PubMed**

Search ((malaria OR plasmodium OR falciparum OR vivax)) AND (health volunteer OR service provider OR staff OR community health worker OR basic health worker OR primary health care provider OR traditional birth attendan* OR auxiliary midwi* OR public health supervisor OR community intervention OR community model)

<https://www.ncbi.nlm.nih.gov/pubmed?term=((malaria%20OR%20plasmodium%20OR%20falciparum%20OR%20vivax))%20AND%20(health%20volunteer%20OR%20service%20provider%20OR%20staff%20OR%20community%20health%20worker%20OR%20basic%20health%20worker%20OR%20primary%20health%20care%20provider%20OR%20traditional%20birth%20attendan*%20OR%20auxiliary%20midwi*%20OR%20public%20health%20supervisor%20OR%20community%20intervention%20OR%20community%20model)>

1. **Embase**

'malaria'/exp OR malaria OR 'plasmodium'/exp OR plasmodium OR falciparum OR vivax AND ('health volunteer' OR 'service provider' OR 'staff'/exp OR staff OR 'community health worker'/exp OR 'community health worker' OR 'basic health worker' OR 'primary health care provider' OR 'traditional birth attendan*' OR 'auxiliary midwi*' OR 'public health supervisor' OR 'community intervention' OR 'community model')

<http://www.embase.com/#advancedSearch/resultspage/history.1/page.1/200.items/orderby.relevance/source>.

1. **Cochrane Central Register of Controlled Trials,**

(malaria OR plasmodium OR falciparum OR vivax)

“AND”

(health volunteer OR service provider OR staff OR community health worker OR basic health worker OR primary health care provider OR traditional birth attendan* OR auxiliary midwi* OR public health supervisor OR community intervention OR community model)

<http://onlinelibrary.wiley.com/cochranelibrary/search>

1. LILACS

(tw:(malaria OR plasmodium OR falciparum OR vivax)) AND (tw:(health-volunteer OR service-provider OR staff OR community-health-worker OR basic-health-worker OR primary-health-care-provider OR traditional-birth-attendan* OR auxiliary-midwi* OR public-health-supervisor OR community-intervention OR community-model))

<http://pesquisa.bvsalud.org/portal/?output=site&lang=en&from=0&sort=RELEVANCE&format=&count=100&fb=&page=1&q=%28tw%3A%28malaria+OR+plasmodium+OR+falciparum+OR+vivax%29%29+AND+%28tw%3A%28health-volunteer+OR+service-provider+OR+staff+OR+community-health-worker+OR+basic-health-worker+OR+primary-health-care-provider+OR+traditional-birth-attendan*+OR+auxiliary-midwi*+OR+public-health-supervisor+OR+community-intervention+OR+community-model%29%29&index=tw>

1. African Medicus Index

malaria OR plasmodium OR falciparum OR vivax [Key Word] and 'health volunteer' OR 'service provider' OR staff OR 'community health worker' OR 'basic health worker' OR 'primary health care provider' OR 'traditional birth attendan*' OR 'auxiliary midwi*' OR 'public health supervisor' OR 'community intervention' OR 'community model' [Key Word]

<http://indexmedicus.afro.who.int/cgi-bin/wxis.exe/iah/>

**Appendix 2 – 2 steps selection of papers and reporting formats**

Step 1 – preliminary screening by title and abstracts (peer reviewed papers) and title, list of content (grey literature)

Step 2 – Final screening by full text

Reporting format for included papers

| **No** | **Title** | **Author** | **Year of publication** | **Journal** | **Objectives addressed** | | | | **Malaria (or) malaria plus  other** | **Type of community delivered model** |
| --- | --- | --- | --- | --- | --- | --- | --- | --- | --- | --- |
|  |  |  |  |  | **1** | **2** | **3** | **4** | **M/O** |  |
| 1 |  |  |  |  |  |  |  |  |  |  |
| 2 |  |  |  |  |  |  |  |  |  |  |
| 3 |  |  |  |  |  |  |  |  |  |  |
| 4 |  |  |  |  |  |  |  |  |  |  |
| 5 |  |  |  |  |  |  |  |  |  |  |

Reporting format for excluded papers after second screening

| **No** | **Title** | **Author** | **Year of Publication** | **Reason for exclusion** |
| --- | --- | --- | --- | --- |
| 1 |  |  |  |  |
| 2 |  |  |  |  |
| 3 |  |  |  |  |
| 4 |  |  |  |  |
| 5 |  |  |  |  |

**Appendix 3 – Data Extraction Structure**

| Basic study information, type of community-delivered model, participants and malaria interventions and additional service/s | | | | | | | | | Integration intervention, service delivery option/s, nature of models | | | | | | | | Outcomes including coverage and achievement by indicators, other uptake measure | | | | | | | | | | | | | | | | | |
| --- | --- | --- | --- | --- | --- | --- | --- | --- | --- | --- | --- | --- | --- | --- | --- | --- | --- | --- | --- | --- | --- | --- | --- | --- | --- | --- | --- | --- | --- | --- | --- | --- | --- | --- |
|  |  |  |  |  |  |  |  |  | Interventions delivered | | Service delivery option/s | | Nature of model | | | | community model | | Coverage | | | | Malaria matric indices | | | | Barriers and Enablers | | | | | | | |
| No | Title | Author | date of Publication | Study design | control (if any) | Population and location | Timing of intervention/study | Malaria transmission structure  (if described) | malaria intervention delivered | Non-malaria interventions delivered | routine program/project | additional for the research purpose | Community engagement, education,  health promotion | Commodities, supply chain, logistics | Health information, reporting and  monitoring | Others: HR planning, policy development, financing and payment schemes | Type of community-delivered model | Different name of Community Health Workers | number and/or percentage, or other measures of service uptake of malaria | number and/or percentage, or other measures of service uptake of other diseases interventions | Indirect malaria related outcomes | Indirect non-malaria related outcomes | Number and/or percentage of people diagnosed with malaria by Rapid Diagnostic Test (RDT), microscopy or Polymerase Chain Reaction (PCR) (malaira infection) | Number and/or percentage of malaria cases | Number and/or percentage of people died from malaria | Other indices | Feasibility or acceptability | CHW knowledge, quality and adherance to guidelines | Costs | Impact on organization of services | Supply chains system and management | Health system issues in the background context | Social or political constraints | Any other barrier or enabler |
| 1 |  |  |  |  |  |  |  |  |  |  |  |  |  |  |  |  |  |  |  |  |  |  |  |  |  |  |  |  |  |  |  |  |  |  |
| 2 |  |  |  |  |  |  |  |  |  |  |  |  |  |  |  |  |  |  |  |  |  |  |  |  |  |  |  |  |  |  |  |  |  |  |
| 3 |  |  |  |  |  |  |  |  |  |  |  |  |  |  |  |  |  |  |  |  |  |  |  |  |  |  |  |  |  |  |  |  |  |  |
| 4 |  |  |  |  |  |  |  |  |  |  |  |  |  |  |  |  |  |  |  |  |  |  |  |  |  |  |  |  |  |  |  |  |  |  |
| 5 |  |  |  |  |  |  |  |  |  |  |  |  |  |  |  |  |  |  |  |  |  |  |  |  |  |  |  |  |  |  |  |  |  |  |

**Appendix 4 – Narrative reporting of qualitative data synthesis**

| Background | Getting Started | Main theme of the narrative |
| --- | --- | --- |
|  |  | Contents included in the narrative |
|  |  | The way it can be used |
|  | Prioritizing topics  (barriers and enablers) | Topics to be considered |
|  |  | Criteria used to set priorities based on the findings |
|  |  | People participated in the priorities setting |
|  |  | Process used in the priorities setting |
| Preparing the narrative | Clarifying the topics  (barriers and enablers ) | What is the topic(s) and how did it come to attention? |
|  |  | How has the problem (topic) been framed (described) and what are the consequences/advantages of this framing? |
|  |  | How big is the topic/problem? |
|  |  | What is the cause of the problem? |
|  | Deciding on the  describing the options | Which options should be presented to address the barriers and to augment the enablers? |
|  |  | What is known about the impacts of different options? |
|  |  | How confident can we be about the likely impacts of each of the options ? |
|  |  | Summary information about the potential impacts of the different options |
|  | Identifying and addressing the barriers to implementing the options | What barriers are there for implementing each option? |
|  |  | What strategies are available to address important barriers ? |
|  |  | What is known about the effects of relevant implementation strategies ? |
|  |  | Summary information about the barriers and likely effects of strategies for addressing them |
|  | Clarifying uncertainties and needs for monitoring and evaluation | Important uncertainties that should be addressed prior to making a decision |
|  |  | Monitoring and evaluation for the progress |
