# Supplementary material for: The impact of community-delivered models of malaria control and elimination: a systematic review
Source: Malar J. 2019 Aug 6;18:269. doi: 10.1186/s12936-019-2900-1 (PMC6683427; doi:10.1186/s12936-019-2900-1)
Supplement: Supplementary file 3 — Additional file 3. Search strategy, syntax and source of grey literature searches. [file 12936_2019_2900_MOESM3_ESM.docx]

**Additional material 3: search strategy, syntax and source of grey literature searches**

1. **PubMed**

Search ((malaria OR plasmodium OR falciparum OR vivax)) AND (health volunteer OR service provider OR staff OR community health worker OR basic health worker OR primary health care provider OR traditional birth attendan* OR auxiliary midwi* OR public health supervisor OR community intervention OR community model)

<https://www.ncbi.nlm.nih.gov/pubmed?term=((malaria%20OR%20plasmodium%20OR%20falciparum%20OR%20vivax))%20AND%20(health%20volunteer%20OR%20service%20provider%20OR%20staff%20OR%20community%20health%20worker%20OR%20basic%20health%20worker%20OR%20primary%20health%20care%20provider%20OR%20traditional%20birth%20attendan*%20OR%20auxiliary%20midwi*%20OR%20public%20health%20supervisor%20OR%20community%20intervention%20OR%20community%20model)>

1. **Embase**

'malaria'/exp OR malaria OR 'plasmodium'/exp OR plasmodium OR falciparum OR vivax AND ('health volunteer' OR 'service provider' OR 'staff'/exp OR staff OR 'community health worker'/exp OR 'community health worker' OR 'basic health worker' OR 'primary health care provider' OR 'traditional birth attendan*' OR 'auxiliary midwi*' OR 'public health supervisor' OR 'community intervention' OR 'community model')

<http://www.embase.com/#advancedSearch/resultspage/history.1/page.1/200.items/orderby.relevance/source>.

1. **Cochrane Central Register of Controlled Trials,**

(malaria OR plasmodium OR falciparum OR vivax)

“AND”

(health volunteer OR service provider OR staff OR community health worker OR basic health worker OR primary health care provider OR traditional birth attendan* OR auxiliary midwi* OR public health supervisor OR community intervention OR community model)

<http://onlinelibrary.wiley.com/cochranelibrary/search>

1. **LILACS**

(tw:(malaria OR plasmodium OR falciparum OR vivax)) AND (tw:(health-volunteer OR service-provider OR staff OR community-health-worker OR basic-health-worker OR primary-health-care-provider OR traditional-birth-attendan* OR auxiliary-midwi* OR public-health-supervisor OR community-intervention OR community-model))

<http://pesquisa.bvsalud.org/portal/?output=site&lang=en&from=0&sort=RELEVANCE&format=&count=100&fb=&page=1&q=%28tw%3A%28malaria+OR+plasmodium+OR+falciparum+OR+vivax%29%29+AND+%28tw%3A%28health-volunteer+OR+service-provider+OR+staff+OR+community-health-worker+OR+basic-health-worker+OR+primary-health-care-provider+OR+traditional-birth-attendan*+OR+auxiliary-midwi*+OR+public-health-supervisor+OR+community-intervention+OR+community-model%29%29&index=tw>

1. **African Medicus Index**

malaria OR plasmodium OR falciparum OR vivax [Key Word] and 'health volunteer' OR 'service provider' OR staff OR 'community health worker' OR 'basic health worker' OR 'primary health care provider' OR 'traditional birth attendan*' OR 'auxiliary midwi*' OR 'public health supervisor' OR 'community intervention' OR 'community model' [Key Word]

<http://indexmedicus.afro.who.int/cgi-bin/wxis.exe/iah/>

**Sources of grey literature searches**

- Government agencies (Ministries of Health, Ministry of Health and Sport, Ministry of Public Health) in areas at risk of malaria as defined by the Malaria Atlas Project;
- UN agencies (World Health Organization <http://www.who.int/en/> , UNICEF <http://www.unicef.org/> , UNOPS <https://www.unops.org/english/Pages/Home.aspx> );
- International Organizations (IO) and Non-Government Organizations (NGO) (Population Services International <http://www.psi.org/> , Malaria Consortium <http://www.malariaconsortium.org/>
- International Organizations for Migration <http://www.iom.int/> , Save the Children International <https://www.savethechildren.net/> , Médecins Sans Frontières (MSF) International <http://www.msf.org/> , Community Partners International <http://cpintl.org/> );
- Philanthropies and donor agencies (Bill and Malinda Gate Foundation <http://www.gatesfoundation.org/> , United States Agency for International Development <https://www.usaid.gov/> , UK Department for International Development <https://www.gov.uk/government/organisations/department-for-international-development> , Australian Department of Foreign Affairs and Trade <http://dfat.gov.au/pages/default.aspx> , Asia Development Bank <http://www.adb.org/> , Japan International Cooperation Agency <http://www.jica.go.jp/english/> ).
