# Supplementary material for: The impact of community-delivered models of malaria control and elimination: a systematic review
Source: Malar J. 2019 Aug 6;18:269. doi: 10.1186/s12936-019-2900-1 (PMC6683427; doi:10.1186/s12936-019-2900-1)
Supplement: Supplementary file 4 — Additional file 4. Papers excluded after second step screening with reasons for exclusion. [file 12936_2019_2900_MOESM4_ESM.docx]

**Additional material 4: papers excluded after second step screening with reasons**

| **No** | **Title** | **Author** | **Date of Publication** |
| --- | --- | --- | --- |
| 1. **Reason for exclusion:** Case series, no new data, opinion pieces, reviews, editorials, conference abstracts, study protocols, posters | | | |
| 1 | National scale-up of integrated community case management in rural Ethiopia: implementation and early lessons learned | | 2014 |
| 2 | Effectiveness of Transfluthrin-Coated Inflammable-Fumes Insecticide-Paper (Rambo) in the prevention of malaria in Kano, Nigeria | Y. M. Abdulsalam, H. Muhammad, A. Abduljalal, Z. Iliyasu, B. M. Muhammad, M. M. Bello, I. A. Sadiq, M. Ma'Arouf and A. G. Habib | 2014 |
| 3 | Eave curtains: Entomological evaluation and community knowledge, attitudes and perceptions in prevention of malaria mosquito entry into human dwellings in kisian and rota villages, kisumu county, Western Kenya | B. O. Abong'o | 2013 |
| 4 | Cost drivers of household treatment of presumptive malaria in home-based management of malaria in Ejisu-Juaben Municipality | P. Agyei-Baffour and B. O. Asante | 2012 |
| 5 | Role of rapid diagnostic testing (RDT) in the context of home management of childhood fever (HMCF) with dispersible artemether-lumefantrine: An open label randomized controlled trial in a rural and seasonal malaria transmission area of Burkina Faso | T. B. Alfred, K. T. Amadou, C. Nathalie, C. Edmond and P. Franco | 2010 |
| 6 | Compliance with malaria chemoprophylaxis over a five-year period among children in a rural area of The Gambia | S. J. Allen, R. W. Snow, A. Menon and B. M. Greenwood | 1990 |
| 7 | Assessing the impact of integrated community case management (iCCM) programs on child mortality: Review of early results and lessons learned in sub-Saharan Africa | A. Amouzou, S. Morris, L. H. Moulton and D. Mukanga | 2014 |
| 8 | The effect of an enhanced antenatal care package for prevention of malaria and anemia in pregnancy in Ghana | G. D. Antwi, H. K. Tagbor and I. Bates | 2014 |
| 9 | Empowering village health teams, a value addition to health services delivery in resource limited settings; case study of kiboga and kyankwanzi districts in uganda | E. M. Asiimwe, F. Kabikira, D. Kayiwa and S. Thiam | 2012 |
| 10 | Piloting integrated community case management of malaria, pneumonia and diarrhea in private sector drug shops in Uganda | P. Awor, H. Wamani, G. Bwire, G. Jagoe and S. Peterson | 2012 |
| 11 | Volunteer Community Health Workers: Temporary Fix Or Long-Term Solution? | K. Banek, J. Nankabirwa, D. DiLiberto, L. Taaka, C. I. Chandler and S. G. Staedke | 2010 |
| 12 | Community based malaria control in Saraya, Southeastern Senegal | D. Blanas and Y. Ndiaye | 2010 |
| 13 | Can volunteer community health workers in rural Uganda provide integrated community case management? | J. L. Brenner, C. Barigye, S. Maling, J. Kabakyenga, A. Nettel-Aguirre, D. Buchner, T. K. Yomuhangi, K. Wotton, N. Amon and N. Singhal | 2014 |
| 14 | Feasibility studies prior to the implementation of artemisinin-based combination therapy for the home management of malaria in children below five years | D. Cameroon, N. D. Shey, E. N. J. Bosco, N. A. B. Prosper, T. P. Ntiamah, T. M. Ngwe and P. Francois | 2010 |
| 15 | The impact of intermittent parasite clearance on malaria, anaemia, and cognition in schoolchildren: New evidence from an area of highly seasonal transmission | S. Clarke, S. Rouhani, S. Diarra, M. Bamadio, R. Jones, D. Traore, M. Jukes, J. Thuilliez, M. Sacko, S. Brooker and N. Roschnik | 2013 |
| 16 | Seasonal malaria chemoprevention and micronutrient supplementation in early childhood: Effect on asymptomatic parasitaemia, anemia and Cognition | S. E. Clarke, Y. Griffiths, Y. Dicko, M. Bamadio, S. Diarra, P. Thera, R. Jones, D. Traore, M. Traore, B. Maiga, A. Dicko, M. Sacko and N. Roschnik | 2014 |
| 17 | Intermittent parasite clearance in schoolchildren: Impact on cognition in an area of highly seasonal transmission | S. E. Clarke, S. Rouhani, S. Diarra, M. Bamadio, R. Jones, D. Traore, M. C. Jukes, J. Thuillez, M. Sacko, S. Brooker, S. Lee and N. Roschnik | 2013 |
| 18 | Seasonal malaria chemoprevention combined with micronutrient supplementation delivered through community preschools: Findings from a cluster randomized trial in Mali | S. E. Clarke, M. Sacko, N. Roschnik, Y. Dicko, S. Diarra, P. Thera, M. Bamadio, R. Saye, R. Jones, Y. Griffiths, L. Pisani, M. Coulibaly, D. Toure, A. Dicko and B. Maiga | 2015 |
| 19 | Evaluation of topical repellents as additional vector control measures to control residual transmission in malaria pre-elimination areas | M. Coosemans, V. Sluydts, S. Tho, S. Siv, H. Somony, L. Canier, K. Nimol, K. Soarin, S. Mao, C. Gryseels, U. Sambunny, K. Roey, K. Kerkhof, K. P. Grietens, D. Menard and L. Durnez | 2014 |
| 20 | Comparison of mortality between villages with and without Primary Health Care workers in Upper River Division, The Gambia | A. De Francisco, J. A. Schellenberg, A. J. Hall, A. M. Greenwood, K. Cham and B. M. Greenwood | 1994 |
| 21 | A cluster-randomized trial of targeted control to eliminate malaria in central Senegal: Study design and acceptability of the interventions | A. Diallo, B. Cissé, F. Tairou, E. H. Bâ, O. Sy, J. Gomis, C. Sokhna, J. Gaudart, C. Flach, O. Gaye, O. Faye and P. Milligan | 2014 |
| 22 | Antimalarial treatment by health care providers in Port Harcourt, Nigeria | O. O. Ebong, E. O. Asuquo, C. A. Nwauche, I. M. Siminialayi, I. H. Ogbuehi and M. F. Ajienka | 2012 |
| 23 | Assessing the effectiveness of household-level focal mass drug administration and community-wide mass drug administration for reducing malaria parasite infection prevalence and incidence in Southern Province, Zambia: Study protocol for a community randomized controlled trial | T. P. Eisele, K. Silumbe, T. Finn, V. Chalwe, M. Kamuliwo, B. Hamainza, H. Moonga, A. Bennett, J. Yukich, J. Keating, R. W. Steketee and J. M. Miller | 2015 |
| 24 | Comparative effectiveness of congregation- versus clinic-based approach to prevention of mother-to-child HIV transmission: study protocol for a cluster randomized controlled trial | E. E. Ezeanolue, M. C. Obiefune, W. Yang, S. K. Obaro, C. O. Ezeanolue and G. G. Ogedegbe | 2013 |
| 25 | Community-based malaria control in Tigray, northern Ethiopia | T. A. Ghebreyesus, K. H. Witten, A. Getachew, K. O'Neill, A. Bosman and A. Teklehaimanot | 1999 |
| 26 | The community-based malaria control programme in Tigray, northern Ethiopia. A review of programme set-up, activities, outcomes and impact | T. A. Ghebreyesus, K. H. Witten, A. Getachew, A. M. Yohannes, W. Tesfay, M. Minass, A. Bosman and A. Teklehaimanot | 2000 |
| 27 | Lack of household clustering of malaria in a complex humanitarian emergency: implications for active case detection | H. Hamze, R. Charchuk, M. K. Jean Paul, K. M. Claude, M. Léon and M. T. Hawkes | 2016 |
| 28 | Experiences engaging community health workers to provide maternal and newborn health services: Implementation of four programs | J. Haver, W. Brieger, J. Zoungrana, N. Ansari and J. Kagoma | 2015 |
| 29 | Measuring the strength of community case management implementation: Validation of mobile phone interviews with community health workers in Malawi | E. Hazel, A. Amouzou, L. Park, B. Banda, T. Chimuna, T. Guenther, H. Nsona, C. Victora and J. Bryce | 2013 |
| 30 | Self-protection from malaria vectors in Pakistan: an evaluation of popular existing methods and appropriate new techniques in Afghan refugee communities | S. E. Hewitt, M. Farhan, H. Urhaman, N. Muhammad, M. Kamal and M. W. Rowland | 1996 |
| 31 | Supervising community health workers in low-income countries--a review of impact and implementation issues | Z. Hill, M. Dumbaugh, L. Benton, K. Källander, D. Strachan, A. ten Asbroek, J. Tibenderana, B. Kirkwood and S. Meek | 2014 |
| 32 | Impact of malaria rapid diagnostic tests on patients' subsequent treatment-seeking, costs and health outcomes: Results from the ACT Consortium | H. Hopkins, P. West, S. Yeung and C. I. Chandler | 2015 |
| 33 | Community perceptions and practices towards malaria control measures in Rwanda: A descriptive qualitative study | C. M. Ingabire, A. Rulisa, J. Alaii, E. Hakizimana, F. Kateera, C. Muvunyi, L. Mutesa and B. Van Den Borne | 2014 |
| 34 | Impact of the availability of integrated community case management on health care seeking behavior in rural Zambia | H. Iyer, P. Seidenberg, D. Hamer, P. Pilingana, K. Sialeeze, K. Semrau and K. Yeboah-Antwi | 2011 |
| 35 | Best practices and innovations with potential to increase coverage of integrated community case management of common childhood illness - Uganda and Mozambique | K. Källander, A. Nanyonjo, Z. Hill, G. Ten Asbroek, S. Meek, B. Kirkwood and J. Tibenderana | 2010 |
| 36 | Evaluating the effect of innovative motivation and supervision approaches on community health worker performance and retention in Uganda and Mozambique: Study protocol for a randomised controlled trial | K. Kallander, D. Strachan, S. Soremekun, Z. Hill, R. Lingam, J. Tibenderana, F. Kasteng, A. Vassall, S. Meek and B. Kirkwood | 2015 |
| 37 | Inscale cluster randomized trial evaluating the effect of innovative motivation and supervision approaches on community health worker performance and retention in uganda and mozambique: Intervention design | K. Kallander, J. Tibenderana, B. Kirkwood, Z. Hill, D. Strachan, S. Soremekun, R. Lingam, A. Vassal, F. Kasteng and S. Meek | 2012 |
| 38 | Malaria chemoprophylaxis to pregnant women provided by community health workers in Saradidi, Kenya. I. Reasons for non-acceptance | D. C. Kaseje, E. K. Sempebwa and H. C. Spencer | 1987 |
| 39 | Usage of community-based chloroquine treatment for malaria in Saradidi, Kenya | D. C. Kaseje, H. C. Spencer and E. K. Sempebwa | 1987 |
| 40 | Addressing artemisinin-resistant malaria by identifying malaria hotspots in Cambodia | S. T. Kheang | 2014 |
| 41 | Reducing malaria among migrants and mobile workers in the greater mekong sub-region by broadening opportunities for malaria services and prevention | S. T. Kheang | 2014 |
| 42 | Increasing access to early malaria diagnosis and prompted treatment in remote cambodian villages | S. T. Kheang, S. Duong and A. Olkkonen | 2011 |
| 43 | A randomized longitudinal factorial design to assess malaria vector control and disease management interventions in rural Tanzania | R. A. Kramer, L. E. Mboera, K. Senkoro, A. Lesser, E. H. Shayo, C. J. Paul and M. L. Miranda | 2014 |
| 44 | The role of community health workers in malaria control in the Philippines | T. R. Lariosa | 1992 |
| 45 | Internally displaced human resources for health: villager health worker partnerships to scale up a malaria control programme in active conflict areas of eastern Burma | C. I. Lee, L. S. Smith, E. K. Shwe Oo, B. C. Scharschmidt, E. Whichard, T. Kler, T. J. Lee and A. K. Richards | 2009 |
| 46 | Factors associated with the use of community health workers in the management of uncomplicated malaria among children in rural Cameroon | F. B. Mbezo and B. A. Nkoum | 2015 |
| 47 | Intermittent preventive treatment of malaria in pregnancy: the effect of new delivery approaches on access and compliance rates in Uganda | A. K. Mbonye, P. Magnussen and I. B. Bygbjerg | 2007 |
| 48 | Effects of introducing malaria rapid diagnostic tests in drug shops: Findings from the evaluation of a cluster randomised trial in Uganda | A. K. Mbonye, P. Magnussen, E. Hutchinson, K. S. Hansen, S. Lal and S. E. Clarke | 2015 |
| 49 | Effect of a community-based delivery of intermittent preventive treatment of malaria in pregnancy on treatment seeking for malaria at health units in Uganda | A. K. Mbonye, K. Schultz Hansen, I. C. Bygbjerg and P. Magnussen | 2008 |
| 50 | Malaria chemoprophylaxis, infection of the placenta and birth weight in Gambian primigravidae | C. Menendez, J. Todd, P. L. Alonso, S. Lulat, N. Francis and B. M. Greenwood | 1994 |
| 51 | Utilization of village health workers within a primary health care programme in The Gambia | A. Menon | 1991 |
| 52 | Sustained protection against mortality and morbidity from malaria in rural Gambian children by chemoprophylaxis given by village health workers | A. Menon, R. W. Snow, P. Byass, B. M. Greenwood, R. J. Hayes and A. B. N'Jie | 1990 |
| 53 | The COSMIC consortium: Community-based scheduled screening and treatment of malaria in pregnancy for improved maternal and infant health | P. Mens and H. Schallig | 2013 |
| 54 | Common illnesses identified by community health workers in the households and referrred to primary health facilities for care | D. M. Mogere, C. S. Loum and D. Kaseje | 2014 |
| 55 | Community-based delivery of health care: What is the capacity for expanding interventions? | J. I. Nankabirwa, K. Banek, D. DiLiberto, L. Taaka, C. Chandler and S. Staedke | 2010 |
| 56 | Costing a large-scale implementation of intermittent preventive treatment of malaria in children delivered through community health workers in Senegal | M. Ndiaye, C. Pitt, L. Conteh, E. H. Ba, P. Camara, O. Gaye, C. Sokhna, J. L. Ndiaye, J. F. Gomis, B. Cissé and P. Milligan | 2011 |
| 57 | Costing a large-scale implementation of seasonal malaria chemoprevention in children delivered through community health workers in senegal | M. Ndiaye, C. Pitt, L. Conteh, E. H. Ba, O. Sy, P. I. Camara, C. Sokhna, J. L. Ndiaye, J. F. Gomis, B. Cisse, O. Gaye and P. Milligan | 2012 |
| 58 | Pecadom plus: An active, community-based approach to malaria detection and treatment | Y. Ndiaye, A. Linn, S. Gaye, I. Henesse, P. Linn, K. Nordstrom, I. A. Manga, M. Bâ and C. Hedrick | 2014 |
| 59 | Referral from community-based treatment providers: Evidence from a cluster-randomised trial of mRDTs at community level in two areas of high and low transmission in Uganda | R. Ndyomugyenyi, S. Lal, K. Hansen, P. Magnussen and S. Clarke | 2013 |
| 60 | Improved targeting of antimalarial treatment in community-based management of malaria: Evidence from cluster-randomized trials in Uganda | R. Ndyomugyenyi, P. Magnussen, K. S. Hansen, S. Lal, C. I. Chandler, A. K. Mbonye and S. E. Clarke | 2014 |
| 61 | Can community health workers provide quality integrated community management of febrile illnesses: A case study of community health workers in two selected local government areas of akwa ibom state, Nigeria | B. C. Orji, W. R. Brieger, E. Otolorin, J. Nwadike, E. V. Bassey and M. Nkanga | 2012 |
| 62 | Scaling-up malaria rapid diagnostic tests and artemisinin-based combination therapy into integrated community case management sites: Results from two remote and low-resource settings in the democratic republic of Congo | J. Otshudiema, N. Embeke, F. Hernandez, J. Tchofa, C. M. Modiri and F. X. Mwema | 2013 |
| 63 | Impact of community case management of malaria and pneumonia on clinical outcome and rational use of drugs: Results from a multi-country study in Sub-Saharan Africa | F. Pagnoni, D. Mukanga, A. Tiono, T. Anyorigiyia, S. Cousens and G. Barnish | 2011 |
| 64 | Community health workers as an effective channel for delivery of child health interventions: Expanding the knowledge base | L. Paintain, B. Willey, A. Sharkey, J. Kim, V. Buj, J. Webster, D. Schellenberg and N. Ngongo | 2012 |
| 65 | Intervention is not enough: Functional social networks are critical to the success of community level malaria control | L. S. Paintain, L. Gueye, M. G. Sall, A. Hyde, M. Claite, B. Gueye, J. Webster and C. Jones | 2011 |
| 66 | Impact of combining intermittent preventive treatment with home management of malaria in children under ten years, in a rural area of Senegal | T. Roger, C. T. Ndour, B. Faye, J. L. Ndiaye, P. Magnussen, C. Bassene, I. C. Bygbjerb and O. Gaye | 2011 |
| 67 | Community participation in malaria surveillance and treatment I. The Volunteer Collaborator Network of Guatemala | T. K. Ruebush Ii and H. A. Godoy | 1992 |
| 68 | Use of illiterate volunteer workers for malaria case detection and treatment | T. K. Ruebush Ii, R. Zeissig, H. A. Godoy and R. E. Klein | 1990 |
| 69 | Reducing the spread of artemisinin resistant malaria through community-level directly observed therapy in Western of Cambodia | C. Say, S. Nguon, S. L. Dy and K. S. Ty | 2012 |
| 70 | Steps towards malaria elimination: Integrating population-wide test and treat campaigns into malaria control in zambia | K. Silumbe, D. Larsen, B. Hamainza, J. M. Miller, C. Lungu, M. Hawela, J. Chirwa and M. Kamuliwo | 2012 |
| 71 | Home Management of Malaria: An additional effective strategy for the integrated approach for malaria eradication | B. S. Sirima | 2009 |
| 72 | Variation in the quality and cost of treatment for childhood diarrhea, malaria and pneumonia: Community and facility based care in rural Uganda | S. Soremekun, F. Kasteng, R. Lingam, G. T. Asbroek, A. Vassall, B. Kirkwood, A. Nanyonjo, E. Kertho and K. Kallander | 2013 |
| 73 | Impact on mortality and fertility of a community-based malaria control programme in Saradidi, Kenya | H. C. Spencer, D. C. Kaseje, W. H. Mosley, E. K. Sempebwa, A. Y. Huong and J. M. Roberts | 1987 |
| 74 | Effectiveness of community health workers in the prevention of malaria in Machakos, Kenya | D. Stromberg, J. Frederiksen, J. Hruschka, A. Tomedi, M. Mwanthi, B. Skipper and D. Broudy | 2010 |
| 75 | An assessment of the malaria-related knowledge and practices of Tanzania's drug retailers: Exploring the impact of drug store accreditation | R. Thomson, B. Johanes, C. Festo, A. Kalolella, M. Taylor, S. Tougher, Y. Ye, A. Mann, R. Ren, K. Bruxvoort, B. Willey, F. Arnold, K. Hanson and C. Goodman | 2014 |
| 76 | Combining community case management of malarial and seasonal malaria chemoprevention for children less than 10 years in Senegal: Feasibility, impact on malaria and Anemia | R. C. Tine, B. Faye, C. T. Ndour, B. Cisse, M. Cairns, M. Ndiaye, J. L. Ndiaye, P. Magnussen, I. Bygbjerg, B. Greenwood and O. Gaye | 2014 |
| 77 | Feasibility, safety and effectiveness of combining home based malaria management (HMM) and seasonal malaria chemoprevention (SMC) in children less than ten years in Senegal: A clusterrandomized trial | R. C. Tine, C. T. Ndour, B. Faye, M. Ndiaye, K. Sylla, D. Sow, J. L. Ndiaye and O. Gaye | 2013 |
| 78 | Evaluating the impact of interventions on malaria trends in the Andean Region | D. Vargas, A. Rosas, B. Pineda, K. Tobar and O. Feo | 2009 |
| 79 | Community based malaria elimination efforts in southern Zambia | A. M. Winters, Z. Chisha, D. Bridges, B. Winters, B. Hamainza, M. Mwanza, M. Kamuliwo, S. Wamulume, D. Earle and J. Miller | 2013 |
| 80 | Effect of integrated community case management of common childhood illnesses on the quality of malaria case management provided by health extension workers at health posts | A. Wogi, D. Teno, T. Bulto, W. Deressa, H. Alemu and M. Nigussie | 2014 |
| 81 | Management of pneumonia and malaria at the community level in Zambia | K. Yeboah-Antwi, P. Pilingana, W. B. MacLeod, K. Semrau, K. Siazeele, P. Kalesha, B. Hamainza, E. Mtonga, P. Chanda, L. Sabin, K. Kamholz, E. Twohig, D. M. Thea and D. H. Hamer | 2009 |
| 82 | Developing the national community health assistant strategy in Zambia: A policy analysis | J. M. Zulu, J. Kinsman, C. Michelo and A. K. Hurtig | 2013 |
| 83 | Systematic literature review of integrated community case management and the private sector in Africa: Relevant experiences and potential next steps | P. Awor, J. Miller and S. Peterson | 2014 |
| 84 | Thirty years after Alma-Ata: a systematic review of the impact of community health workers delivering curative interventions against malaria, pneumonia and diarrhoea on child mortality and morbidity in sub-Saharan Africa | J. B. Christopher, A. Le May, S. Lewin and D. A. Ross | 2011 |
| 85 | Effectiveness of community health workers delivering preventive interventions for maternal and child health in low- and middle-income countries: a systematic review | B. Gilmore and E. McAuliffe | 2013 |
| 86 | Barriers and facilitators to the implementation of lay health worker programmes to improve access to maternal and child health: qualitative evidence synthesis | C. Glenton, J. Colvin Christopher, B. Carlsen, A. Swartz, S. Lewin, J. Noyes and A. Rashidian | 2013 |
| 87 | Impact of home-based management of malaria on health outcomes in Africa: a systematic review of the evidence | H. Hopkins, A. Talisuna, C. J. Whitty and S. G. Staedke | 2007 |
| 88 | Scaling-up malaria treatment: a review of the performance of different providers | M. M. Kamal-Yanni, J. Potet and P. M. Saunders | 2012 |
| 89 | Home- or community-based programmes for treating malaria | C. I. Okwundu, S. Nagpal, A. Musekiwa and D. Sinclair | 2013 |
| 90 | Community health workers and stand-alone or integrated case management of malaria: A systematic literature review | L. S. Paintain, B. Willey, S. Kedenge, A. Sharkey, J. Kim, V. Buj, J. Webster, D. Schellenberg and N. Ngongo | 2014 |
| 91 | Success or failure of critical steps in community case management of malaria with rapid diagnostic tests: a systematic review | E. Ruizendaal, S. Dierickx, K. Peeters Grietens, H. D. Schallig, F. Pagnoni and P. F. Mens | 2014 |
| 92 | Impact of community-based interventions for the prevention and control of malaria on intervention coverage and health outcomes for the prevention and control of malaria (Provisional abstract) | R. A. Salam, J. K. Das, Z. S. Lassi and Z. A. Bhutta | 2014 |
| 1. **Reason for exclusion:** Did not address study objectives | | | |
| 93 | An integrated approach of community health worker support for HIV/AIDS and TB care in Angónia district, Mozambique | S. Simon, K. Chu, M. Frieden, B. Candrinho, N. Ford, H. Schneider and M. Biot | 2009 |
| 94 | Impact of ministry of health interventions on private medicine retailer knowledge and practices on anti-malarial treatment in Kenya | T. Abuya, G. Fegan, Y. Rowa, B. Karisa, S. Ochola, W. Mutemi and V. Marsh | 2009 |
| 95 | The amount and value of work time of community medicine distributors in community case management of malaria among children under five years in the Ejisu-Juaben District of Ghana | P. Agyei-Baffour, K. S. Hansen, E. N. Browne and P. Magnussen | 2012 |
| 96 | Addressing anemia in women and children in rural communities of Cambodia and Kenya: Experiences from an integrated program | B. A. Aidam, A. Edward, A. C. Paden, R. Y. S. Wong and J. Chege | 2016 |
| 97 | A malaria control trial using insecticide-treated bed nets and targeted chemoprophylaxis in a rural area of The Gambia, west Africa. 5. Design and implementation of the trial | P. L. Alonso, S. W. Lindsay, J. R. Armstrong Schellenberg, M. Konteh, K. Keita, C. Marshall, A. Phillips, K. Cham and B. M. Greenwood | 1993 |
| 98 | Effectiveness of supportive supervision on the consistency of integrated community cases management skills of the health extension workers in 113 districts of Ethiopia | A. Ameha, A. M. Karim, A. Erbo, A. Ashenafi, M. Hailu, B. Hailu, A. Folla, S. Bizuwork and W. Betemariam | 2014 |
| 99 | Patient related factors affecting adherence to antimalarial medication in an urban estate in ghana | A. O. Amponsah, H. Vosper and A. F. A. Marfo | 2015 |
| 100 | Cstock-a simple, affordable mhealth solution for improving visibility of community health logistics data | S. Andersson, Y. Chandani, A. Misomali, B. Chimphanga, M. Shieshia, M. Noel and B. Felling | 2013 |
| 101 | Improving the quality of paediatric malaria diagnosis and treatment by rural providers in Myanmar: an evaluation of a training and support intervention | T. Aung, K. Longfield, N. M. Aye, A. K. San, T. S. Sutton and D. Montagu | 2015 |
| 102 | Unequal treatment access and malaria risk in a community-based intervention program in the Philippines | D. Bell, R. Go, C. Miguel, W. Parks and J. Bryan | 2005 |
| 103 | Cluster randomized trial of text message reminders to retail staff in tanzanian drug shops dispensing artemether-lumefantrine: effect on dispenser knowledge and patient adherence | K. Bruxvoort, C. Festo, A. Kalolella, M. Cairns, P. Lyaruu, M. Kenani, S. P. Kachur, C. Goodman and D. Schellenberg | 2014 |
| 104 | Rosie the spray team leader, expanding opportunities for women in malaria prevention | J. Burnett, J. Coleman and N. De Gier | 2014 |
| 105 | Community case management of malaria using ACT and RDT in two districts in Zambia: achieving high adherence to test results using community health workers | P. Chanda, B. Hamainza, H. B. Moonga, V. Chalwe and F. Pagnoni | 2011 |
| 106 | Community health workers use malaria rapid diagnostic tests (RDTs) safely and accurately: results of a longitudinal study in Zambia | H. Counihan, S. A. Harvey, M. Sekeseke-Chinyama, B. Hamainza, R. Banda, T. Malambo, F. Masaninga and D. Bell | 2012 |
| 107 | Evaluation of community-based systems for the surveillance of day three-positive Plasmodium falciparum cases in Western Cambodia | J. Cox, L. Dy Soley, T. Bunkea, S. Sovannaroth, K. Soy Ty, S. Ngak, S. Bjorge, P. Ringwald, S. Mellor, D. Sintasath and S. Meek | 2014 |
| 108 | Mystery shopping in community drug shops: Research as development in rural Tanzania | A. Dillip, S. Alba, C. Mshana, M. Hetzel, J. Liana, C. Lengeler, I. Mayumana, A. Schulze, H. Mshinda and B. Obrist | 2012 |
| 109 | Comparison of a mobile phone-based malaria reporting system with source participant register data for capturing spatial and temporal trends in epidemiological indicators of malaria transmission collected by community health workers in rural Zambia | B. Hamainza, G. F. Killeen, M. Kamuliwo, A. Bennett and J. O. Yukich | 2015 |
| 110 | The health benefits of social mobilization: experiences with community-based Integrated Management of Childhood Illness in Chao, Peru and San Luis, Honduras | T. Harkins, C. Drasbek, J. Arroyo and M. McQuestion | 2008 |
| 111 | Determinants of prompt and adequate care among presumed malaria cases in a community in eastern Rwanda: A cross sectional study | C. M. Ingabire, F. Kateera, E. Hakizimana, A. Rulisa, C. Muvunyi, P. Mens, C. J. M. Koenraadt, L. Mutesa, M. Van Vugt, B. Van Den Borne and J. Alaii | 2016 |
| 112 | The acceptability mass administrations of anti-malarial drug as part of targeted malaria elimination in villages along the Thai-Myanmar border | L. Kajeechiwa, M. M. Thwin, P. W. Shee, N. L. Yee, E. Elvina, P. Peapah, K. Kyawt, P. T. Oo, W. PoWah, J. R. Min, J. Wiladphaingern, L. von Seidlein, S. Nosten and F. Nosten | 2016 |
| 113 | Integrated community case management of malaria and pneumonia increases prompt and appropriate treatment for pneumonia symptoms in children under five years in Eastern Uganda | J. N. Kalyango, T. Alfven, S. Peterson, K. Mugenyi, C. Karamagi and E. Rutebemberwa | 2013 |
| 114 | High adherence to antimalarials and antibiotics under integrated community case management of illness in children less than five years in eastern Uganda | J. N. Kalyango, E. Rutebemberwa, C. Karamagi, E. Mworozi, S. Ssali, T. Alfven and S. Peterson | 2013 |
| 115 | The effect of an anti-malarial subsidy programme on the quality of service provision of artemisinin-based combination therapy in Kenya: a cluster-randomized, controlled trial | B. P. Kangwana, S. V. Kedenge, A. M. Noor, V. A. Alegana, A. J. Nyandigisi, J. Pandit, G. W. Fegan, J. E. Todd, R. W. Snow and C. A. Goodman | 2013 |
| 116 | Oral artemisinin monotherapy removal from the private sector in Eastern Myanmar between 2012 and 2014 | H. S. S. Khin, T. Aung, A. Thi and C. White | 2016 |
| 117 | Malaria community health workers in Myanmar: a cost analysis | S. S. Kyaw, T. Drake, A. Thi, M. P. Kyaw, T. Hlaing, F. M. Smithuis, L. J. White and Y. Lubell | 2016 |
| 118 | Insights from community case management data in six sub-saharan African countries | Y. B. Laínez, A. Wittcoff, A. I. Mohamud, P. Amendola, H. B. Perry and E. D'Harcourt | 2012 |
| 119 | Deployment of community health workers across rural sub-Saharan Africa: financial considerations and operational assumptions | G. C. McCord, A. Liu and P. Singh | 2013 |
| 120 | Malaria rapid testing by community health workers is effective and safe for targeting malaria treatment: randomised cross-over trial in Tanzania | M. Mubi, A. Janson, M. Warsame, A. Mårtensson, K. Källander, M. G. Petzold, B. Ngasala, G. Maganga, L. L. Gustafsson, A. Massele, G. Tomson, Z. Premji and A. Björkman | 2011 |
| 121 | SMS photograph-based external quality assessment of reading and interpretation of malaria rapid diagnostic tests in the Democratic Republic of the Congo | P. Mukadi, P. Gillet, B. Barbé, J. Luamba, A. Lukuka, J. Likwela, D. Mumba, J.-J. Muyembe, P. Lutumba and J. Jacobs | 2015 |
| 122 | Primary care training for patent medicine vendors in rural Nigeria | F. O. Oshiname and W. R. Brieger | 1992 |
| 123 | A malaria control trial using insecticide-treated bed nets and targeted chemoprophylaxis in a rural area of The Gambia, West Africa 8. Cost-effectiveness of bed net impregnation alone or combined with chemoprophylaxis in preventing mortality and morbidity from malaria in Gambian children | J. Picard, M. Aikins, P. L. Alonso, J. Schellenberg, B. M. Greenwood and A. Mills | 1993 |
| 124 | Community case management of childhood illness in sub-Saharan Africa - findings from a cross-sectional survey on policy and implementation | K. Rasanathan, M. Muniz, S. Bakshi, M. Kumar, A. Solano, W. Kariuki, A. George, M. Sylla, R. Nefdt, M. Young and T. Diaz | 2014 |
| 125 | Compliance, safety, and effectiveness of fixed-dose artesunate-amodiaquine for presumptive treatment of non-severe malaria in the context of home management of malaria in Madagascar | A. Ratsimbasoa, H. Ravony, J. A. Vonimpaisomihanta, R. Raherinjafy, M. Jahevitra, R. Rapelanoro, J. Rakotomanga, D. Malvy, P. Millet and D. Menard | 2012 |
| 126 | A multifaceted intervention to improve health worker adherence to integrated management of childhood illness guidelines in Benin | A. K. Rowe, F. Onikpo, M. Lama, D. M. Osterholt, S. Y. Rowe and M. S. Deming | 2009 |
| 127 | The influence of observation and setting on community health workers' practices | S. Y. Rowe, M. A. Olewe, D. G. Kleinbaum, J. E. McGowan Jr, D. A. McFarland, R. Rochat and M. S. Deming | 2006 |
| 128 | Use of community health workers for management of malaria and pneumonia in urban and rural areas in eastern Uganda | E. Rutebemberwa, D. Kadobera, S. Katureebe, J. N. Kalyango, E. Mworozi and G. Pariyo | 2012 |
| 129 | Community point distribution of insecticide-treated bed nets and community health worker hang-up visits in rural Zambia: A decision-focused evaluation | P. Wang, A. L. Connor, A. S. Joudeh, J. Steinberg, K. Ndhlovu, M. Siyolwe, B. Ntebeka, B. Chibuye and B. Hamainza | 2016 |
| 130 | Using "mother trainers" for malaria control: the Nigerian experience | I. O. Ajayi, O. O. Kale, O. Oladepo and E. A. Bamgboye | 2006 |
| 131 | Health worker perceptions of integrating mobile phones into community case management of malaria in Saraya, Senegal | D. A. Blanas, Y. Ndiaye, M. MacFarlane, I. Manga, A. Siddiqui, O. Velez, A. S. Kanter, K. Nichols and N. Hennig | 2015 |
| 132 | Stakeholders' perceptions of integrated community case management by community health workers: a post-intervention qualitative study | D. L. Buchner, J. L. Brenner, J. Kabakyenga, K. Teddy, S. Maling, C. Barigye, A. Nettel-Aguirre and N. Singhal | 2014 |
| 133 | Relative costs and effectiveness of treating uncomplicated malaria in two rural districts in Zambia: implications for nationwide scale-up of home-based management | P. Chanda, B. Hamainza, H. B. Moonga, V. Chalwe, P. Banda and F. Pagnoni | 2011 |
| 134 | Community perceptions on malaria and care-seeking practices in endemic Indian settings: Policy implications for the malaria control programme | A. Das, R. D. Gupta, J. Friedman, M. M. Pradhan, C. C. Mohapatra and D. Sandhibigraha | 2013 |
| 135 | The effect of household heads training on long-lasting insecticide-treated bed nets utilization: a cluster randomized controlled trial in Ethiopia | A. Deribew, Z. Birhanu, L. Sena, T. Dejene, A. A. Reda, M. Sudhakar, F. Alemseged, F. Tessema, A. Zeynudin, S. Biadgilign and K. Deribe | 2012 |
| 136 | Cost effectiveness and resource allocation of Plasmodium falciparum malaria control in Myanmar: a modelling analysis of bed nets and community health workers | T. L. Drake, S. S. Kyaw, M. P. Kyaw, F. M. Smithuis, N. P. Day, L. J. White and Y. Lubell | 2015 |
| 137 | A tool box for operational mosquito larval control: Preliminary results and early lessons from the Urban Malaria Control Programme in Dar es Salaam, Tanzania | U. Fillinger, K. Kannady, G. William, M. J. Vanek, S. Dongus, D. Nyika, Y. Geissbühler, P. P. Chaki, N. J. Govella, E. M. Mathenge, B. H. Singer, H. Mshinda, S. W. Lindsay, M. Tanner, D. Mtasiwa, M. C. De Castro and G. F. Killeen | 2008 |
| 138 | The effects of malaria chemoprophylaxis given by traditional birth attendants on the course and outcome of pregnancy | B. M. Greenwood, A. M. Greenwood, R. W. Snow, P. Byass, S. Bennett and A. B. Hatib-N'Jie | 1989 |
| 139 | Quality and safety of integrated community case management of malaria using rapid diagnostic tests and pneumonia by community health workers | D. H. Hamer, E. T. Brooks, K. Semrau, P. Pilingana, W. B. MacLeod, K. Siazeele, L. L. Sabin, D. M. Thea and K. Yeboah-Antwi | 2012 |
| 140 | Valuing the work of unpaid community health workers and exploring the incentives to volunteering in rural Africa | F. Kasteng, S. Settumba, K. Kallander and A. Vassall | 2016 |
| 141 | Factors associated with utilization of community health workers in improving access to malaria treatment among children in Kenya | J. Kisia, F. Nelima, D. O. Otieno, K. Kiilu, W. Emmanuel, S. Sohani, K. Siekmans, A. Nyandigisi and W. Akhwale | 2012 |
| 142 | Improving malaria knowledge and practices in rural Myanmar through a village health worker intervention: a cross-sectional study | M. M. Lwin, M. Sudhinaraset, A. K. San and T. Aung | 2014 |
| 143 | Intermittent preventive treatment of malaria in pregnancy: the incremental cost-effectiveness of a new delivery system in Uganda | A. K. Mbonye, K. S. Hansen, I. C. Bygbjerg and P. Magnussen | 2008 |
| 144 | Perceived quality of care for common childhood illnesses: Facility versus community based providers in Uganda | A. Nanyonjo, F. Makumbi, P. Etou, G. Tomson and K. Källander | 2013 |
| 145 | Appropriate targeting of artemisinin-based combination therapy by community health workers using malaria rapid diagnostic tests: findings from randomized trials in two contrasting areas of high and low malaria transmission in south-western Uganda | R. Ndyomugyenyi, P. Magnussen, S. Lal, K. Hansen and S. E. Clarke | 2016 |
| 146 | Distribution of Subsidized Insecticide-treated Bed Nets through a Community Health Committee in Boboye Health District, Niger | D. Nonaka, A. Maazou, S. Yamagata, I. Oumarou, T. Uchida, H. J. Yacouba, J. Kobayashi, T. Takeuchi and T. Mizoue | 2012 |
| 147 | Inequities in valuation of benefits, choice of drugs, and mode of payment for malaria treatment services provided by community health workers in Nigeria | O. Onwujekwe, J. Ojukwu, E. Shu and B. Uzochukwu | 2007 |
| 148 | Feasibility of a community health worker strategy for providing near and appropriate treatment of malaria in southeast Nigeria: an analysis of activities, costs and outcomes | O. Onwujekwe, B. Uzochukwu, J. Ojukwu, N. Dike and E. Shu | 2007 |
| 149 | Attitudes toward home-based malaria testing in rural and urban Sierra Leone | S. Ranasinghe, R. Ansumana, J. M. Lamin, A. S. Bockarie, U. Bangura, J. A. G. Buanie, D. A. Stenger and K. H. Jacobsen | 2015 |
| 150 | Qualities of an ideal volunteer community malaria worker: a comparison of the opinions of community residents and national malaria service staff | T. K. Ruebush, 2nd, S. C. Weller and R. E. Klein | 1994 |
| 151 | Reduced paediatric hospitalizations for malaria and febrile illness patterns following implementation of community-based malaria control programme in rural Rwanda | A. C. Sievers, J. Lewey, P. Musafiri, M. F. Franke, B. J. Bucyibaruta, S. N. Stulac, M. L. Rich, C. Karema and J. P. Daily | 2008 |
| 152 | Acceptability by community health workers in Senegal of combining community case management of malaria and seasonal malaria chemoprevention | R. C. Tine, P. Ndiaye, C. T. Ndour, B. Faye, J. L. Ndiaye, K. Sylla, M. Ndiaye, B. Cisse, D. Sow, P. Magnussen, I. C. Bygbjerg and O. Gaye | 2013 |
| 153 | Increases in correct administration of chloroquine in the home and referral of sick children to health facilities through a community-based intervention in Bougouni District, Mali | P. J. Winch, A. Bagayoko, A. Diawara, M. Kané, F. Thiéro, K. Gilroy, Z. Daou, Z. Berthé and E. Swedberg | 2003 |
| 154 | Community case management of fever due to malaria and pneumonia in children under five in Zambia: a cluster randomized controlled trial | K. Yeboah-Antwi, P. Pilingana, W. B. Macleod, K. Semrau, K. Siazeele, P. Kalesha, B. Hamainza, P. Seidenberg, A. Mazimba, L. Sabin, K. Kamholz, D. M. Thea and D. H. Hamer | 2010 |
| 155 | Evaluation of a volunteer community-based health worker program for providing contraceptive services in Madagascar | M. F. Gallo, J. Walldorf, R. Kolesar, A. Agarwal, A. P. Kourtis, D. J. Jamieson and A. Finlay | 2013 |
| 156 | Integrated management of childhood illness (IMCI) strategy for children under five | T. Gera, D. Shah, P. Garner, M. Richardson and S. Sachdev Harshpal | 2016 |
| 157 | Lay health workers in primary and community health care for maternal and child health and the management of infectious diseases | S. Lewin, S. Munabi-Babigumira, C. Glenton, K. Daniels, X. Bosch-Capblanch, E. van Wyk Brian, J. Odgaard-Jensen, M. Johansen, N. Aja Godwin, M. Zwarenstein and B. Scheel Inger | 2010 |
| 158 | Impact of community-based maternal health workers on coverage of essential maternal health interventions among internally displaced communities in eastern Burma: the MOM project | L. C. Mullany, T. J. Lee, L. Yone, C. I. Lee, K. C. Teela, P. Paw, E. K. Shwe Oo, C. Maung, H. Kuiper, N. F. Masenior and C. Beyrer | 2010 |
| 159 | Impact of a positive deviance approach to improve the effectiveness of an iron-supplementation program to control nutritional anemia among rural Senegalese pregnant women | M. Ndiaye, K. Siekmans, S. Haddad and O. Receveur | 2009 |
| 160 | A Cross-Sectional Study of Community-Based Maternal and Child Health Interventions Involving Women's Health Volunteer Groups in Rural Myanmar | M. Oguro and S. Horiuchi | 2016 |
| 161 | Selling Sprinkles micronutrient powder reduces anemia, iron deficiency, and vitamin A deficiency in young children in Western Kenya: a cluster-randomized controlled trial | P. S. Suchdev, L. J. Ruth, B. A. Woodruff, C. Mbakaya, U. Mandava, R. Flores-Ayala, M. E. Jefferds and R. Quick | 2012 |
| 162 | Health facility caseload changes during the introduction of community case management of malaria in South Western Uganda - An interrupted time series approach | S. Lal, R. Ndyomugyenyi, M. Lagarde, N. D. Alexander, L. Paintain, P. Magnussen, D. Chandramohan and S. E. Clarke | 2015 |
| 163 | Factors related to retention of community health workers in a trial on community-based management of fever in children under 5 years in the Dangme West District of Ghana | M. Abbey, L. K. Bartholomew, J. Nonvignon, M. A. Chinbuah, M. Pappoe, M. Gyapong, J. O. Gyapong, C. Bart-Plange and B. van den Borne | 2014 |
| 164 | Sustainability of intervention for home management of malaria: The Nigerian experience | I. O. Ajayi, S. A. Jegede and C. O. Falade | 2010 |
| 165 | Effects of the integrated Community Case Management of Childhood Illness Strategy on Child Mortality in Ethiopia: A Cluster Randomized Trial | A. Amouzou, E. Hazel, B. Shaw, N. P. Miller, M. Tafesse, Y. Mekonnen, L. H. Moulton, J. Bryce and R. E. Black | 2016 |
| 166 | Assessment of knowledge and factors that may predict willingness to volunteerism: a pilot study of community-directed distributors in Anambra state | S. U. Arinze-Onyia, I. Modebe, E. N. Aguwa and E. Nwobodo | 2015 |
| 167 | Performance of community health workers managing malaria, pneumonia and diarrhoea under the community case management programme in central Uganda: a cross sectional study | J. Bagonza, S. P. Kibira and E. Rutebemberwa | 2014 |
| 168 | Community case management of malaria: exploring support, capacity and motivation of community medicine distributors in Uganda | K. Banek, J. Nankabirwa, C. Maiteki-Sebuguzi, D. DiLiberto, L. Taaka, C. I. Chandler and S. G. Staedke | 2015 |
| 169 | Successful integration of insecticide-treated bed net distribution with mass drug administration in Central Nigeria | B. G. Blackburn, A. Eigege, H. Gotau, G. Gerlong, E. Miri, W. A. Hawley, E. Mathieu and F. Richards | 2006 |
| 170 | Barriers to community case management of malaria in Saraya, Senegal: training, and supply-chains | D. A. Blanas, Y. Ndiaye, K. Nichols, A. Jensen, A. Siddiqui and N. Hennig | 2013 |
| 171 | Village malaria worker performance key to the elimination of artemisinin-resistant malaria: a Western Cambodia health system assessment | S. E. Canavati, S. Lawpoolsri, C. E. Quintero, C. Nguon, P. Ly, S. Pukrittayakamee, D. Sintasath, P. Singhasivanon, K. Peeters Grietens and M. A. Whittaker | 2016 |
| 172 | Deployment of early diagnosis and mefloquine-artesunate treatment of falciparum malaria in Thailand: the Tak Malaria Initiative | V. I. Carrara, S. Sirilak, J. Thonglairuam, C. Rojanawatsirivet, S. Proux, V. Gilbos, A. Brockman, E. A. Ashley, R. McGready, S. Krudsood, S. Leemingsawat, S. Looareesuwan, P. Singhasivanon, N. White and F. Nosten | 2006 |
| 173 | Community-owned resource persons for malaria vector control: enabling factors and challenges in an operational programme in Dar es Salaam, United Republic of Tanzania | P. P. Chaki, S. Dongus, U. Fillinger, A. Kelly and G. F. Killeen | 2011 |
| 174 | Relaunch of the official community health worker programme in Mozambique: is there a sustainable basis for iCCM policy? | B. G. Chilundo, J. L. Cliff, A. R. Mariano, D. C. Rodriguez and A. George | 2015 |
| 175 | Do community health workers perceive mechanisms associated with the success of community case management of malaria? A qualitative study from Burkina Faso | T. Druetz, K. Kadio, S. Haddad, S. Kouanda and V. Ridde | 2015 |
| 176 | Utilization of community health workers for malaria treatment: results from a three-year panel study in the districts of Kaya and Zorgho, Burkina Faso | T. Druetz, V. Ridde, S. Kouanda, A. Ly, S. Diabate and S. Haddad | 2015 |
| 177 | Feasibility and acceptability of home-based management of malaria strategy adapted to Sudan's conditions using artemisinin-based combination therapy and rapid diagnostic test | K. A. Elmardi, E. M. Malik, T. Abdelgadir, S. H. Ali, A. H. Elsyed, M. A. Mudather, A. H. Elhassan and I. Adam | 2009 |
| 178 | Integrating child health services into malaria control services of village malaria workers in remote Cambodia: service utilization and knowledge of malaria management of caregivers | A. Hasegawa, J. Yasuoka, P. Ly, C. Nguon and M. Jimba | 2013 |
| 179 | Increased use of community medicine distributors and rational use of drugs in children less than five years of age in Uganda caused by integrated community case management of fever | J. N. Kalyango, A. Lindstrand, E. Rutebemberwa, S. Ssali, D. Kadobera, C. Karamagi, S. Peterson and T. Alfven | 2012 |
| 180 | Introduction of newborn care within integrated community case management in Uganda | C. N. Kayemba, H. N. Sengendo, J. Ssekitooleko, K. Kerber, K. Källander, P. Waiswa, P. Aliganyira, T. Guenther, N. Gamache, C. Strachan, C. Ocan, G. Magumba, H. Counihan, A. K. Mbonye and D. R. Marsh | 2012 |
| 181 | A quasi-experimental evaluation of an interpersonal communication intervention to increase insecticide-treated net use among children in Zambia | J. Keating, P. Hutchinson, J. M. Miller, A. Bennett, D. A. Larsen and B. Hamainza | 2012 |
| 182 | Evaluating active roles of community health workers in accelerating universal access to health services for malaria in Palawan, the Philippines | E. L. A. Matsumoto-Takahashi and S. Kano | 2016 |
| 183 | Nationwide implementation of integrated community case management of childhood illness in Rwanda | C. Mugeni, A. C. Levine, R. M. Munyaneza, E. Mulindahabi, H. C. Cockrell, J. Glavis-Bloom, C. T. Nutt, C. M. Wagner, E. Gaju, A. Rukundo, J. P. Habimana, C. Karema, F. Ngabo and A. Binagwaho | 2014 |
| 184 | Acceptability and Utilization of Community Health Workers after the Adoption of the Integrated Community Case Management Policy in Kabarole District in Uganda | G. Muhumuza, C. Mutesi, F. Mutamba, P. Ampuriire and C. Nangai | 2015 |
| 185 | Access, acceptability and utilization of community health workers using diagnostics for case management of fever in Ugandan children: a cross-sectional study | D. Mukanga, J. K. Tibenderana, S. Peterson, G. W. Pariyo, J. Kiguli, P. Waiswa, R. Babirye, G. Ojiambo, S. Kasasa, F. Pagnoni and K. Kallander | 2012 |
| 186 | Integrated community case management of fever in children under five using rapid diagnostic tests and respiratory rate counting: a multi-country cluster randomized trial | D. Mukanga, A. B. Tiono, T. Anyorigiya, K. Källander, A. T. Konaté, A. R. Oduro, J. K. Tibenderana, L. Amenga-Etego, S. B. Sirima, S. Cousens, G. Barnish and F. Pagnoni | 2012 |
| 187 | Community acceptability and adoption of integrated community case management in Uganda | A. Nanyonjo, M. Nakirunda, F. Makumbi, G. Tomson and K. Källander | 2012 |
| 188 | Effectiveness of artemether-lumefantrine provided by community health workers in under-five children with uncomplicated malaria in rural Tanzania: an open label prospective study | B. E. Ngasala, M. Malmberg, A. M. Carlsson, P. E. Ferreira, M. G. Petzold and D. Blessborn | 2011 |
| 189 | Challenges encountered by local health volunteers in early diagnosis and prompt treatment of malaria in Myanmar artemisinin resistance containment zones | M. H. Nyunt, K. M. Aye, K. T. Kyaw, S. S. Han, T. T. Aye, K. T. Wai and M. P. Kyaw | 2016 |
| 190 | Using community-owned resource persons to provide early diagnosis and treatment and estimate malaria burden at community level in north-eastern Tanzania | A. S. Rutta, F. Francis, B. P. Mmbando, D. S. Ishengoma, S. H. Sembuche, E. K. Malecela, J. Y. Sadi, M. L. Kamugisha and M. M. Lemnge | 2012 |
| 191 | Impact of integrated community case management on health-seeking behavior in rural Zambia | P. D. Seidenberg, D. H. Hamer, H. Iyer, P. Pilingana, K. Siazeele, B. Hamainza, W. B. MacLeod and K. Yeboah-Antwi | 2012 |
| 192 | Access to integrated community case management of childhood illnesses services in rural Ethiopia: a qualitative study of the perspectives and experiences of caregivers | B. Shaw, A. Amouzou, N. P. Miller, M. Tafesse, J. Bryce and P. J. Surkan | 2016 |
| 193 | Integrated community case management of malaria, pneumonia and diarrhoea across three African countries: A qualitative study exploring lessons learnt and implications for further scale up | C. Strachan, A. Wharton-Smith, C. Sinyangwe, D. Mubiru, J. Ssekitooleko, J. Meier, M. Gbanya, J. K. Tibenderana and H. Counihan | 2014 |
| 194 | Using theory and formative research to design interventions to improve community health worker motivation, retention and performance in Mozambique and Uganda | D. L. Strachan, K. Kallander, M. Nakirunda, S. Ndima, A. Muiambo and Z. Hill | 2015 |
| 195 | A community health worker program for the prevention of malaria in eastern Kenya | D. G. Stromberg, J. Frederiksen, J. Hruschka, A. Tomedi and M. Mwanthi | 2011 |
| 196 | Feasibility and implementation of community-based malaria case management with integrated vector control in the Democratic Republic of Congo | E. K. Swana, G. Y. Makan, C. K. Mukeng, H. I. Mupumba, G. M. Kalaba, O. N. Luboya and M. J. Bangs | 2016 |
| 197 | Rapid decrease of malaria morbidity following the introduction of community-based monitoring in a rural area of central Vietnam | N. D. Thang, A. Erhart, X. Hung le, K. Thuan le, N. X. Xa, N. N. Thanh, P. V. Ky, M. Coosemans, N. Speybroeck and U. D'Alessandro | 2009 |
| 1. **Reason for exclusion:** Not community-delivered models | | | |
| 198 | The impact of a hybrid social marketing intervention on inequities in access, ownership and use of insecticide-treated nets | S. Agha, R. Van Rossem, G. Stallworthy and T. Kusanthan | 2007 |
| 199 | Impact of health education intervention on insecticide treated nets uptake among nursing mothers in rural communities in Nigeria | O. E. Amoran, K. O. Fatugase, O. M. Fatugase and K. O. Alausa | 2012 |
| 200 | Effect of the Integrated Management of Childhood Illness strategy on childhood mortality and nutrition in a rural area in Bangladesh: a cluster randomised trial | S. E. Arifeen, D. M. Hoque, T. Akter, M. Rahman, M. E. Hoque, K. Begum, E. K. Chowdhury, R. Khan, L. S. Blum, S. Ahmed, M. A. Hossain, A. Siddik, N. Begum, Q. Sadeq-ur Rahman, T. M. Haque, S. M. Billah, M. Islam, R. A. Rumi, E. Law, Z. A. Al-Helal, A. H. Baqui, J. Schellenberg, T. Adam, L. H. Moulton, J. P. Habicht, R. W. Scherpbier, C. G. Victora, J. Bryce and R. E. Black | 2009 |
| 201 | Community effectiveness of intermittent preventive treatment for infants (IPTi) in rural southern Tanzania | J. R. Armstrong Schellenberg, K. Shirima, W. Maokola, F. Manzi, M. Mrisho, A. Mushi, H. Mshinda, P. Alonso, M. Tanner and D. M. Schellenberg | 2010 |
| 202 | Supportive supervision: an effective intervention in achieving high quality malaria case management at primary health care level in Jos, Nigeria | D. A. Bello, Z. I. Hassan, T. O. Afolaranmi, Y. O. Tagurum, O. O. Chirdan and A. I. Zoakah | 2013 |
| 203 | Effect of deploying community health assistants on appropriate treatment for diarrhoea, malaria and pneumonia: quasi-experimental study in two districts of Zambia | G. Biemba, K. Yeboah-Antwi, K. B. Vosburg, M. L. Prust, B. Keller, Y. Worku, H. Zulu, E. White and D. H. Hamer | 2016 |
| 204 | Managerial supervision to improve primary health care in low- and middle-income countries | X. Bosch-Capblanch, S. Liaqat and P. Garner | 2011 |
| 205 | The Impact of Hotspot-Targeted Interventions on Malaria Transmission in Rachuonyo South District in the Western Kenyan Highlands: A Cluster-Randomized Controlled Trial | T. Bousema, G. Stresman, A. Y. Baidjoe, J. Bradley, P. Knight, W. Stone, V. Osoti, E. Makori, C. Owaga, W. Odongo, P. China, S. Shagari, O. K. Doumbo, R. W. Sauerwein, S. Kariuki, C. Drakeley, J. Stevenson and J. Cox | 2016 |
| 206 | Knowledge of medicine outlets staff and their practices for prevention and management of malaria in Ghana | K. O. Buabeng, L. Matowe, F. Smith, M. Duwiejua and H. Enlund | 2009 |
| 207 | Alphamethrin-impregnated bed nets for malaria and mosquito control in China | L. Dapeng, L. Deling, Y. Renguo, L. Peng, H. Xueguang, L. Aimin, W. Lei, G. Changyin, Z. Shaowen, H. Hongru and S. Leyuan | 1994 |
| 208 | Effect of combining mosquito repellent and insecticide treated net on malaria prevalence in Southern Ethiopia: A cluster-randomised trial | W. Deressa, Y. Y. Yihdego, Z. Kebede, E. Batisso, A. Tekalegne and G. A. Dagne | 2014 |
| 209 | Effectiveness of post-campaign, door-to-door, hang-up, and communication interventions to increase long-lasting, insecticidal bed net utilization in Togo (2011-2012): A cluster randomized, control trial | R. E. Desrochers, K. Siekmans, P. R. Berti, K. Bramhill, S. A. Buchan, G. K. Battah, D. Gbetoglo, K. Vignikin and A. Sabino | 2014 |
| 210 | Child malaria in sub-saharan Africa: effective control and prevention require a health promotion approach | D. Houeto and A. Deccache | 2007 |
| 211 | Community health worker performance in the management of multiple childhood illnesses: Siaya District, Kenya, 1997-2001 | J. M. Kelly, B. Osamba, R. M. Garg, M. J. Hamel, J. J. Lewis, S. Y. Rowe, A. K. Rowe and M. S. Deming | 2001 |
| 212 | Health education for community-based malaria control: an intervention study in Ecuador, Colombia and Nicaragua | A. Kroeger, R. Meyer, M. Mancheno and M. González | 1996 |
| 213 | Economic evaluation of a cluster randomized trial of interventions to improve health workers' practice in diagnosing and treating uncomplicated malaria in Cameroon (Provisional abstract) | L. Mangham-Jefferies, V. Wiseman, O. A. Achonduh, T. L. Drake, B. Cundill, O. Onwujekwe and W. Mbacham | 2014 |
| 214 | Application of mobile-technology for disease and treatment monitoring of malaria in the &quot;Better Border Healthcare Programme&quot | P. Meankaew, J. Kaewkungwal, A. Khamsiriwatchara, P. Khunthong, P. Singhasivanon and W. Satimai | 2010 |
| 215 | Distribution systems of insecticide-treated bed nets for malaria control in rural Burkina Faso: cluster-randomized controlled trial | O. Müller, M. Allegri, H. Becher, J. Tiendrebogo, C. Beiersmann, M. Ye, B. Kouyate, A. Sie and A. Jahn | 2008 |
| 216 | Effectiveness of Provider and Community Interventions to Improve Treatment of Uncomplicated Malaria in Nigeria: A Cluster Randomized Controlled Trial | O. Onwujekwe, L. Mangham-Jefferies, B. Cundill, N. Alexander, J. Langham, O. Ibe, B. Uzochukwu and V. Wiseman | 2015 |
| 217 | Experience with the use of community health extension workers in primary care, in a private rural health care institution in South-South Nigeria | B. Ordinioha and C. Onyenaporo | 2010 |
| 218 | The effect of an integrated multisector model for achieving the Millennium Development Goals and improving child survival in rural sub-Saharan Africa: A non-randomised controlled assessment | P. M. Pronyk, M. Muniz, B. Nemser, M. A. Somers, L. McClellan, C. A. Palm, U. K. Huynh, Y. B. Amor, B. Begashaw, J. W. McArthur, A. Niang, S. E. Sachs, P. Singh, A. Teklehaimanot and J. D. Sachs | 2012 |
| 219 | Effect of multiple interventions on community health workers' adherence to clinical guidelines in Siaya district, Kenya | S. Y. Rowe, J. M. Kelly, M. A. Olewe, D. G. Kleinbaum, J. E. McGowan Jr, D. A. McFarland, R. Rochat and M. S. Deming | 2007 |
| 220 | The effect of delivery mechanisms on the uptake of bed net re-impregnation in Kilifi District, Kenya | R. W. Snow, E. McCabe, C. N. Mbogo, C. S. Molyneux, E. S. Some, Mung, V. O. ala and C. G. Nevill | 1999 |
| 221 | The clinical impact of combining intermittent preventive treatment with home management of malaria in children aged below 5 years: cluster randomised trial | H. Tagbor, M. Cairns, E. Nakwa, E. Browne, B. Sarkodie, H. Counihan, S. Meek and D. Chandramohan | 2011 |
| 1. **Reason for exclusion:** Not the intended study participant | | | |
| 222 | Global challenges with scale-up of the integrated management of childhood illness strategy: results of a multi-country survey | A. E. Goga and L. M. Muhe | 2011 |
| 223 | Interventions to improve motivation and retention of community health workers delivering integrated community case management (iCCM): Stakeholder perceptions and priorities | D. L. Strachan, K. Källander, A. H. A. Ten Asbroek, B. Kirkwood, S. R. Meek, L. Benton, L. Conteh, J. Tibenderana and Z. Hill | 2012 |
| 224 | The impact of providing rapid diagnostic malaria tests on fever management in the private retail sector in Ghana: a cluster randomized trial | E. K. Ansah, S. Narh-Bana, H. Affran-Bonful, C. Bart-Plange, B. Cundill, M. Gyapong and C. J. Whitty | 2015 |
| 225 | Improving uptake and use of malaria rapid diagnostic tests in the context of artemisinin drug resistance containment in eastern Myanmar: An evaluation of incentive schemes among informal private healthcare providers | T. Aung, C. White, D. Montagu, W. McFarland, T. Hlaing, H. S. S. Khin, A. K. San, C. Briegleb, I. Chen and M. Sudhinaraset | 2015 |
| 226 | Increased access to care and appropriateness of treatment at private sector drug shops with integrated management of malaria, pneumonia and diarrhoea: a quasi-experimental study in Uganda | P. Awor, H. Wamani, T. Tylleskar, G. Jagoe and S. Peterson | 2014 |
| 227 | Introducing malaria rapid diagnostic tests at registered drug shops in Uganda: limitations of diagnostic testing in the reality of diagnosis | C. I. Chandler, R. Hall-Clifford, T. Asaph, M. Pascal, S. Clarke and A. K. Mbonye | 2011 |
| 228 | A pharmacy too far? Equity and spatial distribution of outcomes in the delivery of subsidized artemisinin-based combination therapies through private drug shops | J. M. Cohen, O. Sabot, K. Sabot, M. Gordon, I. Gross, D. Bishop, M. Odhiambo, Y. Ipuge, L. Ward, A. Mwita and C. Goodman | 2010 |
| 229 | Drug shop regulation and malaria treatment in Tanzania - Why do shops break the rules, and does it matter? | C. Goodman, S. P. Kachur, S. Abdulla, P. Bloland and A. Mills | 2007 |
| 230 | 'It puts life in us and we feel big': shifts in the local health care system during the introduction of rapid diagnostic tests for malaria into drug shops in Uganda | E. Hutchinson, C. Chandler, S. Clarke, S. Lal, P. Magnussen, M. Kayendeke, C. Nabirye, J. Kizito and A. Mbonye | 2015 |
| 231 | The impact of retail-sector delivery of artemether-lumefantrine on malaria treatment of children under five in Kenya: a cluster randomized controlled trial | B. P. Kangwana, S. V. Kedenge, A. M. Noor, V. A. Alegana, A. J. Nyandigisi, J. Pandit, G. W. Fegan, J. E. Todd, S. Brooker, R. W. Snow and C. A. Goodman | 2011 |
| 232 | Understanding the Impact of Subsidizing Artemisinin-Based Combination Therapies (ACTs) in the Retail Sector - Results from Focus Group Discussions in Rural Kenya | S. V. Kedenge, B. P. Kangwana, E. W. Waweru, A. J. Nyandigisi, J. Pandit, S. J. Brooker, R. W. Snow and C. A. Goodman | 2013 |
| 233 | Introducing rapid diagnostic tests for malaria into registered drug shops in Uganda: Lessons learned and policy implications | A. K. Mbonye, S. E. Clarke, S. Lal, C. I. Chandler, E. Hutchinson, K. S. Hansen and P. Magnussen | 2015 |
| 234 | Improving access to early treatment of malaria: a trial with primary school teachers as care providers | G. Y. Afenyadu, I. A. Agyepong, G. Barnish and S. Adjei | 2005 |
| 235 | School-based participatory health education for malaria control in Ghana: engaging children as health messengers | I. Ayi, D. Nonaka, J. K. Adjovu, S. Hanafusa, M. Jimba, K. M. Bosompem, T. Mizoue, T. Takeuchi, D. A. Boakye and J. Kobayashi | 2010 |
| 236 | Improving educational achievement and anaemia of school children: design of a cluster randomised trial of school-based malaria prevention and enhanced literacy instruction in Kenya | S. Brooker, G. Okello, K. Njagi, M. M. Dubeck, K. E. Halliday, H. Inyega and M. C. Jukes | 2010 |
