# Supplementary material for: The impact of community-delivered models of malaria control and elimination: a systematic review
Source: Malar J. 2019 Aug 6;18:269. doi: 10.1186/s12936-019-2900-1 (PMC6683427; doi:10.1186/s12936-019-2900-1)
Supplement: Supplementary file 5 — Additional file 5. Quality assessment by ‘Risk Of Bias in Non-randomized Studies—of Interventions (ROBIN-I)’ for Non-randomized studies and by ‘Cochrane Collaboration’s Tool for assessing risk of bias’ for Randomized studies. [file 12936_2019_2900_MOESM5_ESM.docx]

**Additional Material 5: Quality assessment by ‘Risk Of Bias in Non-randomized Studies - of Interventions (ROBIN-I)’ for Non-randomized studies and by the ‘Cochrane Collaboration’s Tool for assessing risk of bias’ for Randomized studies**


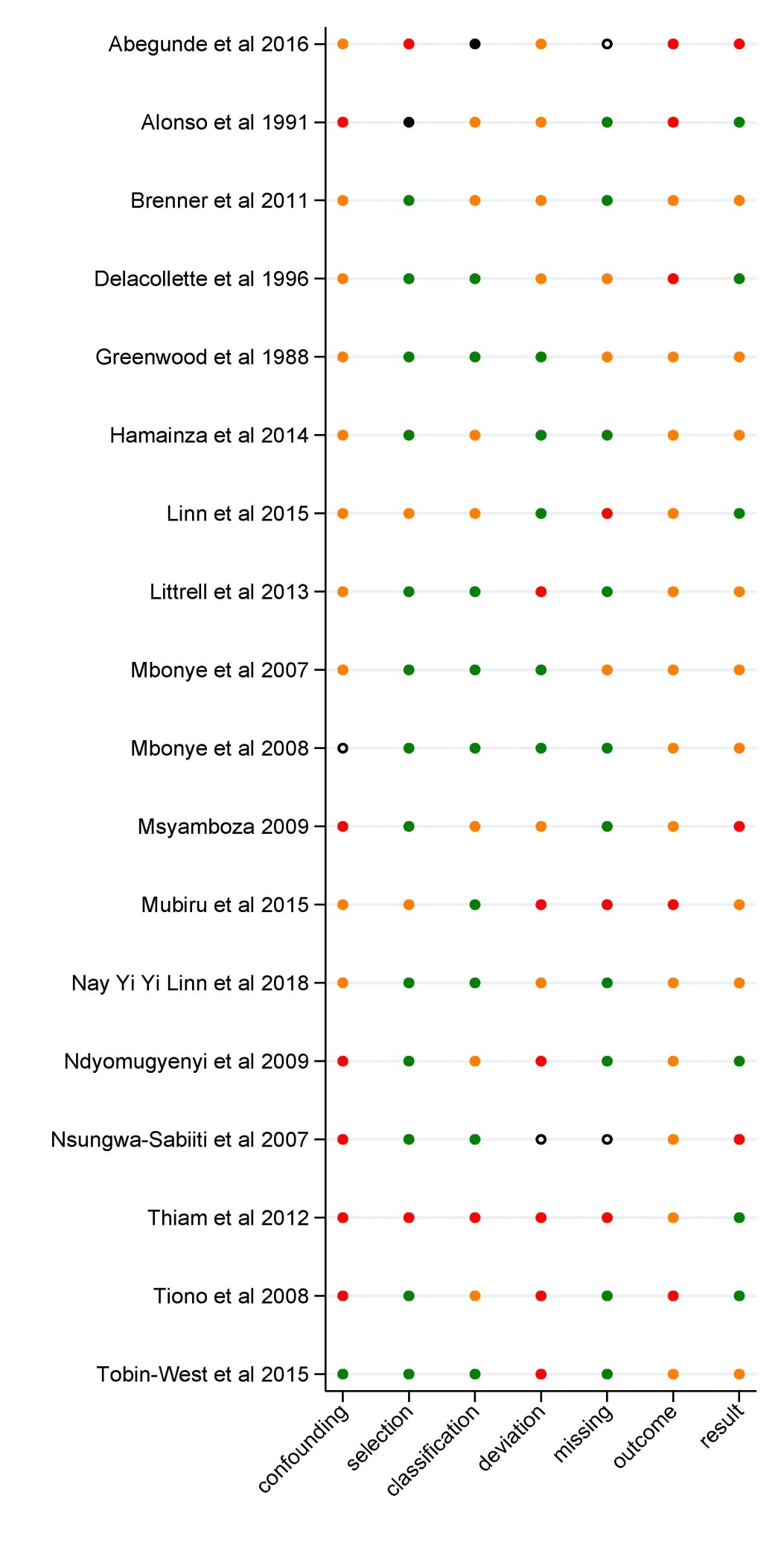


**Figure 1: Quality assessment by ROBIN-I for Non-randomized studies** (green=low risk, orange=moderate risk, red=serious risk, black=critical risk and black circle=no information)

All 18 non-randomized studies (figure 1) had sufficient information to assess bias using ROBIN-I; 6 studies were classified as moderate risk of bias, where 10 studies were seriously biased and 2 were critically biased. The two critically biased papers had critical weaknesses in controlling bias in selection of participants into the study or bias in classification of interventions specified for pre-intervention. ROBINS-I assessment based on the 7 domains of bias and, the tool and detail explanations are available at <https://sites.google.com/site/riskofbiastool/> .

**
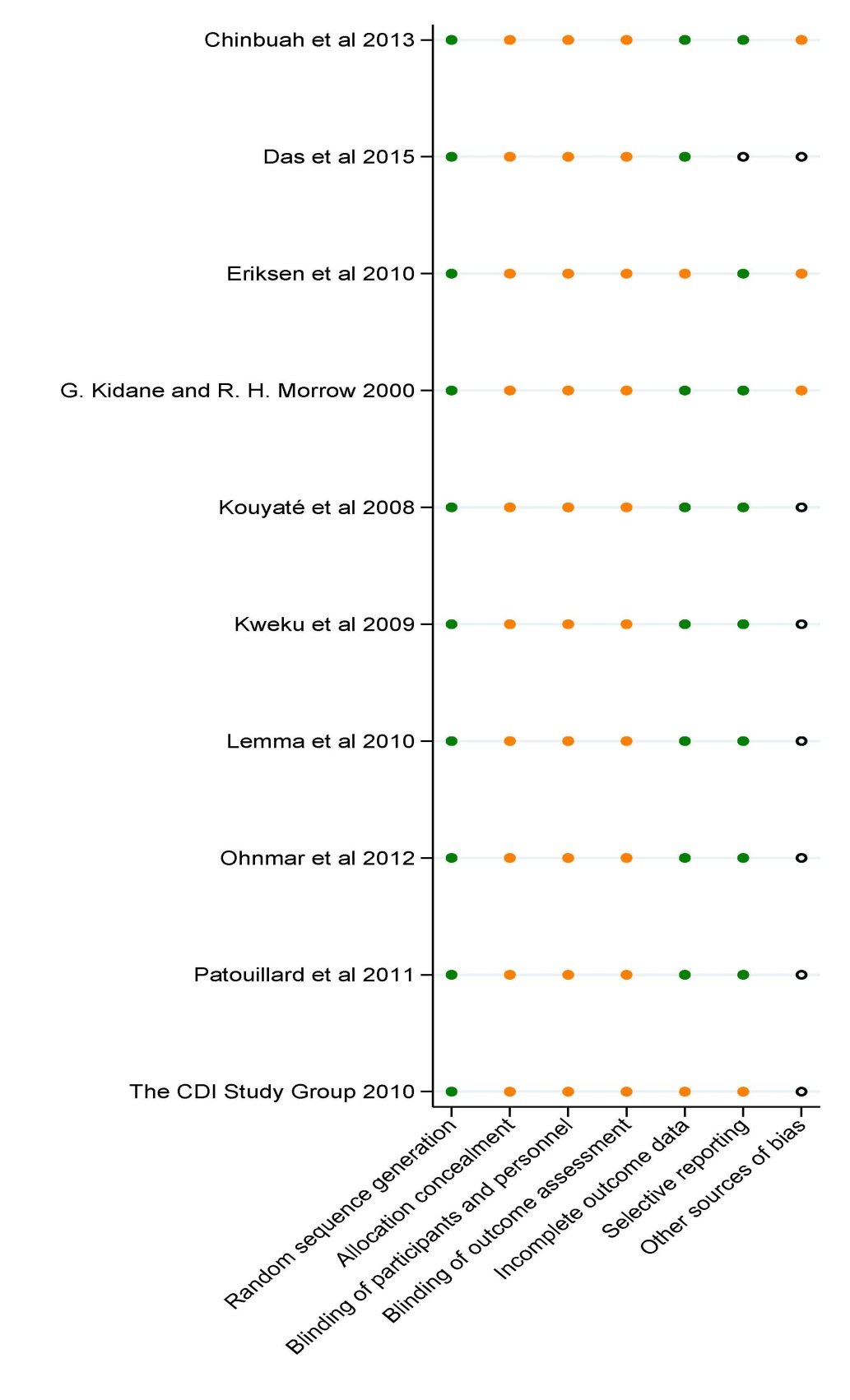
**

**Figure 2: Quality assessment by the Cochrane Collaboration’s Tool for assessing risk of bias: Randomized studies** (green=low risk, orange=moderate risk, red=serious risk, black=critical risk and black circle=no information)

All 10 randomized studies (figure 3) were classified as moderate risk of bias according to the Cochrane assessment tool results for randomized studies. The tool and detail explanations are available at <http://methods.cochrane.org/bias/assessing-risk-bias-included-studies>
